# Comparative genomic analysis of the WRKY III gene family in *populus*, grape, *arabidopsis* and rice

**DOI:** 10.1186/s13062-015-0076-3

**Published:** 2015-09-08

**Authors:** Yiyi Wang, Lin Feng, Yuxin Zhu, Yuan Li, Hanwei Yan, Yan Xiang

**Affiliations:** Laboratory of Modern Biotechnology, School of Forestry and Landscape Architecture, Anhui Agricultural University, Hefei, 230036 China; Key Laboratory of Crop Biology of Anhui Agriculture University, Hefei, 230036 China

**Keywords:** WRKY III, Microsynteny, Gene duplication, Expression, *Populus*

## Abstract

**Background:**

WRKY III genes have significant functions in regulating plant development and resistance. In plant, WRKY gene family has been studied in many species, however, there still lack a comprehensive analysis of WRKY III genes in the woody plant species poplar, three representative lineages of flowering plant species are incorporated in most analyses: *Arabidopsis* (a model plant for annual herbaceous dicots), grape (one model plant for perennial dicots) and *Oryza sativa* (a model plant for monocots).

**Results:**

In this study, we identified 10, 6, 13 and 28 WRKY III genes in the genomes of *Populus trichocarpa*, grape (*Vitis vinifera*), *Arabidopsis thaliana* and rice (*Oryza sativa*), respectively. Phylogenetic analysis revealed that the WRKY III proteins could be divided into four clades. By microsynteny analysis, we found that the duplicated regions were more conserved between poplar and grape than *Arabidopsis* or rice. We dated their duplications by Ks analysis of *Populus* WRKY III genes and demonstrated that all the blocks were formed after the divergence of monocots and dicots. Strong purifying selection has played a key role in the maintenance of WRKY III genes in *Populus*. Tissue expression analysis of the WRKY III genes in *Populus* revealed that five were most highly expressed in the xylem. We also performed quantitative real-time reverse transcription PCR analysis of WRKY III genes in *Populus* treated with salicylic acid, abscisic acid and polyethylene glycol to explore their stress-related expression patterns.

**Conclusions:**

This study highlighted the duplication and diversification of the WRKY III gene family in *Populus* and provided a comprehensive analysis of this gene family in the *Populus* genome. Our results indicated that the majority of WRKY III genes of *Populus* was expanded by large-scale gene duplication. The expression pattern of *PtrWRKYIII* gene identified that these genes play important roles in the xylem during poplar growth and development, and may play crucial role in defense to drought stress. Our results presented here may aid in the selection of appropriate candidate genes for further characterization of their biological functions in poplar.

**Reviewers:**

This article was reviewed by Prof Dandekar and Dr Andrade-Navarro.

**Electronic supplementary material:**

The online version of this article (doi:10.1186/s13062-015-0076-3) contains supplementary material, which is available to authorized users.

## Background

Transcription factors (TFs) are a class of proteins that regulate gene expression in all living organisms. They bind to specific DNA sequences in the promoter regions of genes to activate or repress transcription of multiple target genes. WRKY TFs, are a family of regulatory genes that were first identified in plants [1]. The WRKY TFs, which are important members of the stress-related TF family, are involved in the regulation of plant developmental processes, and in the biotic and abiotic stress response [2]. A common feature of all WRKY TF is the WRKY domain, a highly conserved stretch of about 60 amino acids [3]. Each WRKY domain contains a zinc finger motif at the C-terminus and the strictly conserved amino acid sequence WRKYGQK at its N-terminus [3]. Based on the number of WRKY domains and the pattern of the zinc-finger motif, the WRKY superfamily of plant TFs were classified into three groups (I-III) in *Arabidopsis thaliana* [3], rice (*Oryza sativa*) [4], grape (*Vitis vinifera* [5] and poplar (*Populus trichocarpa)* [6, 7], respectively. WRKY proteins containing a single WRKY domain with C2-H2 pattern belong to group II. Those containing two WRKY domains with C2-H2 pattern are group I. The others, containing a WRKY domain with C2-HC pattern, belong to group III. Group III differs from groups I and II in its altered C2-HC zinc finger motif C-X7-C-X23-HX [3, 8].

Certain WRKY TFs participate in biotic stress responses mediated by hormones, such as jasmonic acid (JA) and salicylic acid (SA) [9, 10], both of which are important defense signals in response to diseases, insects and fungal pathogens [11]. Other WRKY TFs are involved in regulating gene expression in plants during abiotic stresses, such as cold [12, 13], salt [14, 15] and drought [16–18]. Many studies have suggested that WRKY genes participate in the phytohormone abscisic acid (ABA)-mediated drought responses [17].

Although the WRKY gene family has been studied for many years in many species, we know little about the mechanism WRKY gene expansion and the evolutionary forces driving the diversification of this gene family in flowering plants. Poplar WRKY genes were published in 2012 [6] and 2014 [7], making this species a model plant for perennial dicots. And the poplar shows fast growth and can endure adverse environments (abiotic and biotic stresses), including drought. Furthermore, as an ecologically and economically important species, *Populus* is being intensively studied in the light of increasing needs for biofuel production worldwide. In addition, we still lack a comprehensive analysis of group III genes in the woody plant species poplar. Therefore, a study of poplar WRKY III genes would be useful to understand the important biological functions of these genes. The WRKY III genes in flowering plants are thought to have originated after the divergence of the monocots and eudicots [19]. Temporal expression analysis of group III members in *A. thaliana* supported the view that these members are part of different plant defense signaling pathway, including compatible, incompatible, and non-host interactions, indicating their functional differentiation [20]. Thus, the WRKY III genes seem to have played a key role in plant adaption and evolution. The WRKY III genes are considered as the most advanced in terms of evolution, and the most successful in terms of adaptability [19]. Certain WRKY III genes have a significant impact on disease and drought resistance.

In most comparative genomic analysis, three representative lineages of flowering plant species are incorporated in most analysis: *Arabidopsis* (a model plant for annual herbaceous dicots), grape (one model plant for perennial dicots) and *Oryza sativa* (a model plant for monocots). The genomes of *Arabidopsis*, grape and *Oryza sativa* were published in 2003 [20], 2014 [5], and 2005 [4], respectively.

Here, we performed a comparative genomic analysis of the WRKY III gene family in four representative plant species. We reconstructed the phylogenetic tree of this gene family, documented their chromosomal distribution and structural characteristics, explored their conserved microsynteny and gene duplication, assessed the influence of strong purifying selection, and determined expression profiles of *Populus* WRKY III genes in a variety of organs/tissues, and in response to biotic and abiotic stress. Our analysis provided valuable information about WRKY III genes that will aid future functional and ecological studies of this important gene family in flowering plants, especially in *Populus*.

## Results

### Chromosomal distribution and physical properties of WRKY III family in four species genomes

**Fig. 1:**
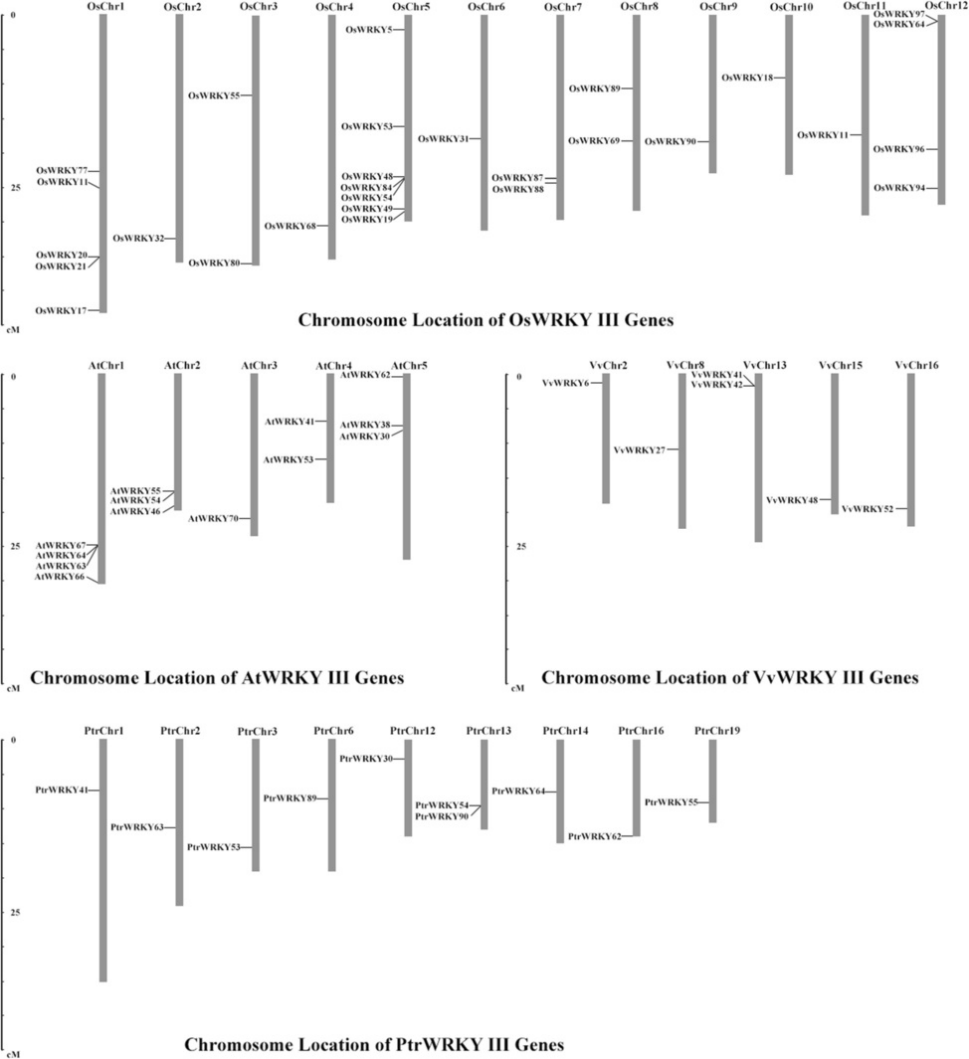
Chromosomal location of WRKY III genes. The distribution of WRKY III genes among the chromosomes in each species is diverse. The chromosome number is indicated at the top of each chromosome

Fifty-seven genes were identified as members of the WRKY III gene family, 13 genes in *Arabidopsis*, 6 genes in grape, 28 genes in rice and 10 genes in *Populus*. Based on these findings, the physical location of individual of WRKY III genes on the chromosomes were determined. The results showed that the 57 WRKY III genes were not evenly distributed on all chromosomes of the four species, as shown in Fig. 1. The genome maps of the WRKY III genes indicated that *OsWRKYIIIs* and *AtWRKYIIIs* were dispersed across all chromosomes, while *VvWRKYIIIs* were distributed on five out of 19 chromosomes (chr 2, 8, 13, 15 and 16), and *PtrWRKYIIIs* were mainly found on nine out of 19 chromosomes (chr 1, 2, 3, 6, 12, 13, 14, 16 and 19). Chromosome 5 contains the most *OsWRKYIII* genes (7), followed by OsChr1(5) and AtChr1 (4). By constrast, the *VvWRKYIIIs* and *PtrWRKYIIIs* were distributed discretely in each chromosome. Among the 57 genes, *OsWRKY90* encodes the longest protein (633 amino acids (aa)), while the shortest (210 aa) was encoded by *OsWRKY55*. The average length of the proteins encoded by the WRKY proteins was 340 aa. The theoretical pI values of the three proteins *(PtrWRKY55*, *AtWRKY41*, *AtWRKY55*) were above 7, indicating that they were alkaline, whereas the proteins encoded by the other WRKY III genes were acidic (<7). Furthermore, the molecular weights of these proteins ranged from 26.4 kDa to 157.6 kDa, with an average of 57.0 kDa. The detailed parameters were shown in Table 1. Although the distribution of these WRKY III genes were diverse, their genetic features and biochemical properties apparently tended toward identify.

**Table 1:** List of WRKY III genes identified in *Populus*, Grape, *Arabidopsis* and Rice, their sequence characteristics

Name	Gene Identifier	Chr	Location COORDINATES (5′- 3′)	ORF length (bp)	Protein			
					Length (a.a.)	PI	Mol.Wt. (Da)	Exons
PtrWRKY41	Potri.001G092900.1	1	7326486 - 7329009	1017	338	6.1	38600.47	3
PtrWRKY63	Potri.002G168700.1	2	12778165 - 12781030	1092	363	6.04	41572.99	3
PtrWRKY53	Potri.003G138600.1	3	15656901 - 15658916	1029	342	5.46	39024.98	3
PtrWRKY89	Potri.006G109100.1	6	8522038 - 8524071	1002	333	6.24	38321.26	3
PtrWRKY30	Potri.012G031700.1	12	2820069 - 2822264	1116	371	5.81	41993.43	3
PtrWRKY90	Potri.013G090400.1	13	9549330 - 9551441	1059	352	5.97	39666.24	3
PtrWRKY54	Potri.013G090300.1	13	9541636 - 9543313	975	324	5.48	37325.53	3
PtrWRKY64	Potri.014G096200.1	14	7526597 - 7529192	1098	365	5.23	41813.27	3
PtrWRKY62	Potri.016G137900.1	16	14049379 - 14051741	966	321	6.06	36678.63	3
PtrWRKY55	Potri.019G059300.1	19	9106420 - 9108112	756	351	7.82	27324.05	3
VvWRKY6	GSVIVT01019511001	2	1228314 - 1229702	1029	342	6.05	38561.86	3
VvWRKY27	GSVIVT01030174001	8	10843756 - 10846082	996	331	5.76	37484.73	5
VvWRKY41	GSVIVT01032662001	13	1716836 - 1718836	927	308	6.72	34255.17	4
VvWRKY42	GSVIVT01032661001	13	1719393 - 1720884	867	288	5.71	32693.34	4
VvWRKY48	GSVIVT01027069001	15	18191021 - 18193489	1083	360	5.16	40234	5
VvWRKY52	GSVIVT01028718001	16	19477141 - 19479868	1095	364	5.45	40043.4	3
AtWRKY30	AT5G24110.1	5	8153115 - 8154709	912	303	6.11	33985.32	3
AtWRKY38	AT5G22570.1	5	7495539 - 7496784	870	289	5.4	33268.25	3
AtWRKY41	AT4G11070.1	4	6759303–6760794	942	313	9.23	34894.21	3
AtWRKY46	AT2G46400.1	2	19043414 - 19044826	888	295	5.73	33634.72	3
AtWRKY53	AT4G23810.1	4	12392370 - 12393982	975	324	6.34	36272.58	2
AtWRKY54	AT2G40750.1	2	17000454 - 17002468	1041	346	5.23	38645.28	3
AtWRKY55	AT2G40740.1	2	16997177 - 16999277	879	292	7.69	32488.79	3
AtWRKY62	AT5G01900.1	5	351008 - 352069	792	263	5.91	30442.52	2
AtWRKY63	AT1G66600.1	1	24848320 - 24849364	726	241	5.63	27378.84	3
AtWRKY64	AT1G66560.1	1	24833579–24834631	750	249	4.89	28549.92	3
AtWRKY66	AT1G80590.1	1	30296210 - 30297156	708	235	5.8	26402.81	3
AtWRKY67	AT1G66550.1	1	24828537 - 24829589	765	254	6.34	29039.72	3
AtWRKY70	AT3G56400.1	3	20908928 - 20910481	885	294	5.85	32935.64	3
OsWRKY77	LOC_Os01g40260.1	1	22731943 - 22733240	741	246	5.05	59691.44	3
OsWRKY11	LOC_Os01g43650.1	1	25009453 - 25012236	1140	379	4.97	92538.29	3
OsWRKY17	LOC_Os01g74140.1	1	42946753 - 42948750	1233	410	4.96	101998.04	3
OsWRKY20	LOC_Os01g60540.1	1	35008866 - 35011098	1128	375	4.99	90787.21	3
OsWRKY21	LOC_Os01g60640.1	1	35062734 - 35064940	843	280	5.03	67914.43	2
OsWRKY32	LOC_Os02g53100.1	2	32489017 - 32495070	1815	604	4.88	145142.97	6
OsWRKY55	LOC_Os03g20550.1	3	11650824 - 11652144	633	210	5.14	52608.57	3
OsWRKY80	LOC_Os03g63810.1	3	36039164 - 36043822	1164	387	4.96	97006.11	3
OsWRKY68	LOC_Os04g51560.1	4	30545175 - 30546577	930	309	4.96	78001.61	3
OsWRKY5	LOC_Os05g04640.1	5	2179520 - 2184940	1509	502	4.89	122971.29	6
OsWRKY53	LOC_Os05g27730.1	5	16150266 - 16152747	1464	487	4.89	119588.82	5
OsWRKY48	LOC_Os05g40060.1	5	23529423 - 23530499	996	331	5	80700.18	2
OsWRKY84	LOC_Os05g40070.1	5	23536113 - 23539013	843	280	5.06	68885.55	3
OsWRKY54	LOC_Os05g40080.1	5	23550611 - 23551716	987	328	5.01	80020.28	2
OsWRKY49	LOC_Os05g49100.1	5	28154693 - 28157989	1260	419	4.95	101051.58	3
OsWRKY19	LOC_Os05g49620.1	5	28471802 - 28473061	834	277	5.04	67350.85	3
OsWRKY31	LOC_Os06g30860.1	6	17915923 - 17917083	1041	346	4.97	84041.8	2
OsWRKY87	LOC_Os07g39480.1	7	23654076 - 23659625	1857	618	4.93	152671	6
OsWRKY88	LOC_Os07g40570.1	7	24311898 - 24315383	1299	432	5	106573.1	4
OsWRKY89	LOC_Os08g17400.1	8	10633195 - 10639603	1653	550	4.99	130711.96	4
OsWRKY69	LOC_Os08g29660.1	8	18220041 - 18222408	960	319	4.97	78415.14	2
OsWRKY90	LOC_Os09g30400.3	9	18496949 - 18500579	1902	633	4.91	157640.64	5
OsWRKY18	LOC_Os10g18099.1	10	9184625 - 9192018	831	276	5.04	68355.38	3
OsWRKY72	LOC_Os11g29870.1	11	17352085 - 17355820	729	242	5.06	59599.38	2
OsWRKY97	LOC_Os12g02420.1	12	802489 - 806097	675	224	5.09	56271.46	3
OsWRKY64	LOC_Os12g02450.1	12	824302 - 825793	966	321	5.04	79376.04	3
OsWRKY96	LOC_Os12g32250.1	12	19473728 - 19478606	1623	540	4.93	133380.3	6
OsWRKY94	LOC_Os12g40570.1	12	25100479 - 25104175	1098	365	5.03	87261.58	4

### Phylogenetic analysis of WRKY III genes in rice, grape, *Arabidopsis* and *Populus*

**Fig. 2:**
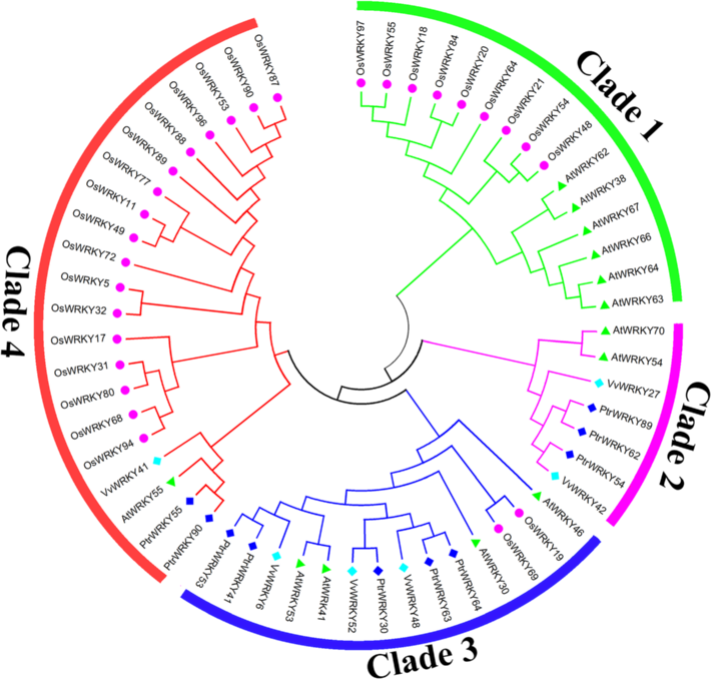
Phylogenetic tree of full-length WRKY III proteins from *Populus*, grape, *Arabidopsis* and rice. The tree was constructed using the neighbor-joining (NJ) method with MEGA 6.0. Dicotyledonous (*Populus*, grape and *Arabidopsis*) and monocotyledonous (rice) WRKY III proteins are marked with colored dots. The tree was also divided into four shared clades (clades 1–4) according to the bootstrap support and evolutionary distances. The gene names are listed in Additional file 1: Table S1

To investigate the similarity and evolutionary ancestry of the WRKY III genes in rice, grape, *Arabidopsis* and *Populus*, we constructed an unrooted phylogenetic tree of the 57 WRKY III protein sequences. The phylogenetic tree was constructed using MEGA 6.0 by employing the Neighbor-Joining (NJ) and Maximum Parsimony (MP) methods, respectively. The tree topologies produced by the two algorithms were largely comparable with only minor modifications at interior branches (data not shown). Therefore, only the NJ phylogenetic tree was subject to further analysis in our study, and the results were completely consistent with previously studies [7]. Bootstrapping tests were performed on these trees. The generated trees were compared and the tree best supported by those methods was used to account for the observations. As indicated in Fig. 2, the WRKY III proteins were divided into four clades by the phylogenetic tree. Clade 2 has the fewest WRKY III gene members (7), while clade 4 contains the most members (21), followed by clade 1 (15) and clade 3 (14). Each of the four species contributed at least one WRKY III gene to clade 3 and clade 4, while the members of the clade 1 and clade 2 included two or three species, for example, clade 1 consisted of rice and *Arabidopsis*, this distribution may correspond to some special events (the split of monocots and dicots) in the evolutionary process. Based on the phylogenetic analysis, two pairs of orthologous genes were identified among the WRKY III genes: *PtrWRKY54* and *VvWRKY42*, and *PtrWRKY30* and *VvWRKY52*. Most genes in the WRKY III gene family are represented by paralogous pairs.

### Gene structure and conserved motifs of WRKY III genes

**Fig. 3:**
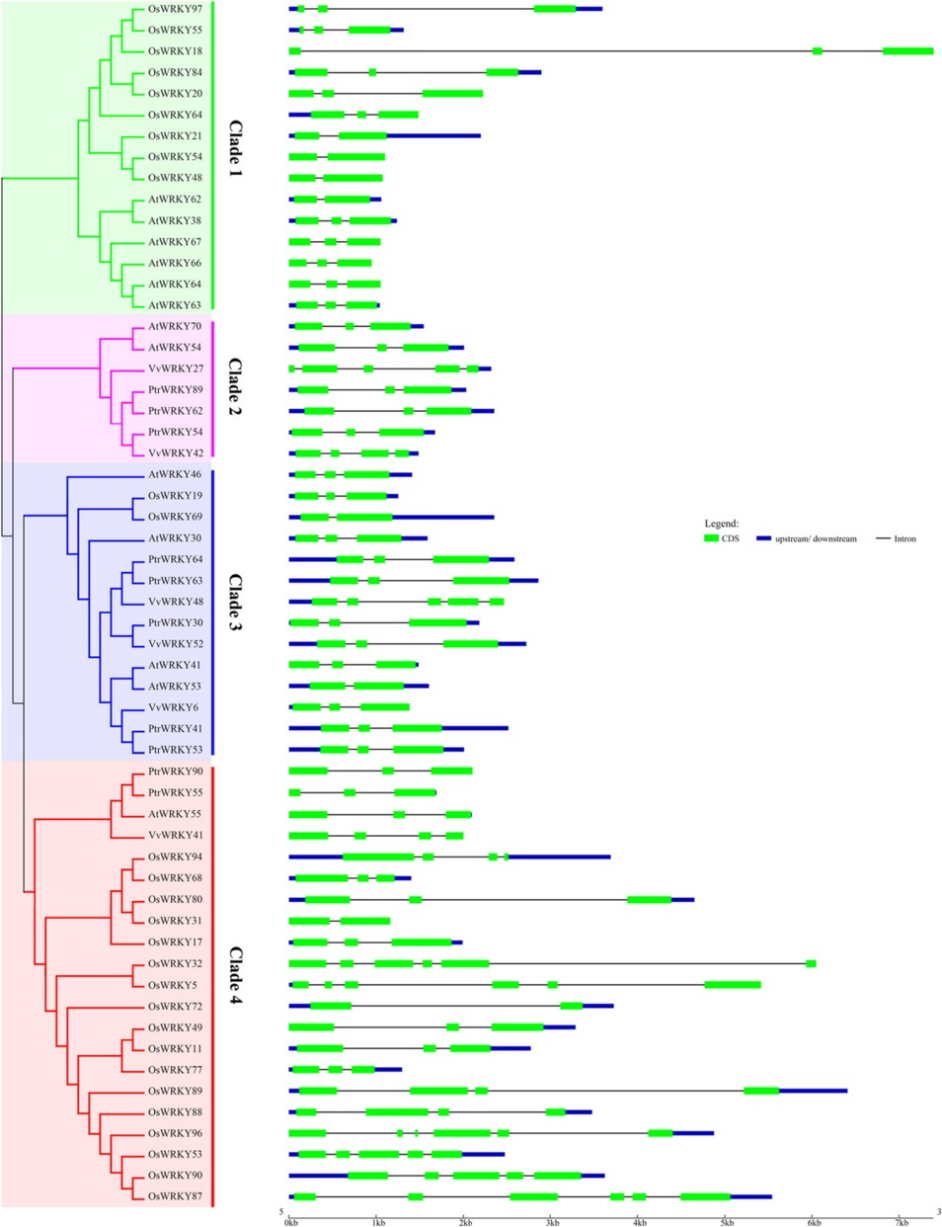
Phylogenetic relationship of WRKY III proteins and the exon-intron structure of WRKY III genes From *Populus*, grape, *Arabidopsis* and rice. Left panel: an unrooted phylogenetic tree constructed using MEGA 6.0 by the N-J method. Clades of WRKY III genes (1–4) are highlighted with different colored backgrounds. Right panel: exon-intron structure. The exons and introns are indicated by green rectangles and thin lines, respectively. The untranslated regions (UTRs) are indicated by thick blue lines

It is well known that gene structural diversity drives the evolution of multigene families. To better understand the structural diversity of WRKY III genes, we generated exon/intron organization maps from the coding sequences of each WRKY III gene. The details structural analysis of the exon/intron were presented in Fig. 3. The 57 WRKY III genes contain different numbers of exons, ranging from 2 to 6. Furthermore, eight WRKY genes were found to possess two exons, thirty-six members had three exons and five had four exons; four genes had five exons and four had six exons. This data indicated that both exon loss and gain has occurred during the evolution of the WRKY III gene family, which may explain the functional diversity of closely related WRKY III genes. We further analyzed the exon/intron structure of the WRKY III orthologous and paralogous gene pairs that clustered together at the terminal branch of the phylogenetic tree to obtain some traceable information about these genes. Among these genes, the exon number of six pairs had changed, including *AtWRKY62/-38*, *PtrWRKY54*/*VvWRKY42*, *AtWRKY41/-53*, *OsWRKY94/-68*, *OsWRKY80/-31*, *OsWRKY90/-87* (Fig. 3). By comparing the six pairs, we found that *AtWRKY62*, *AtWRKY53* and *OsWRKY31* lost one exon during the long evolutionary period, while *VvWRKY42*, *OsWRKY94* and *OsWRKY87* gained one exon. These differences may have been derived from single intron loss or gain events during the long evolutionary period.

**Fig. 4:**
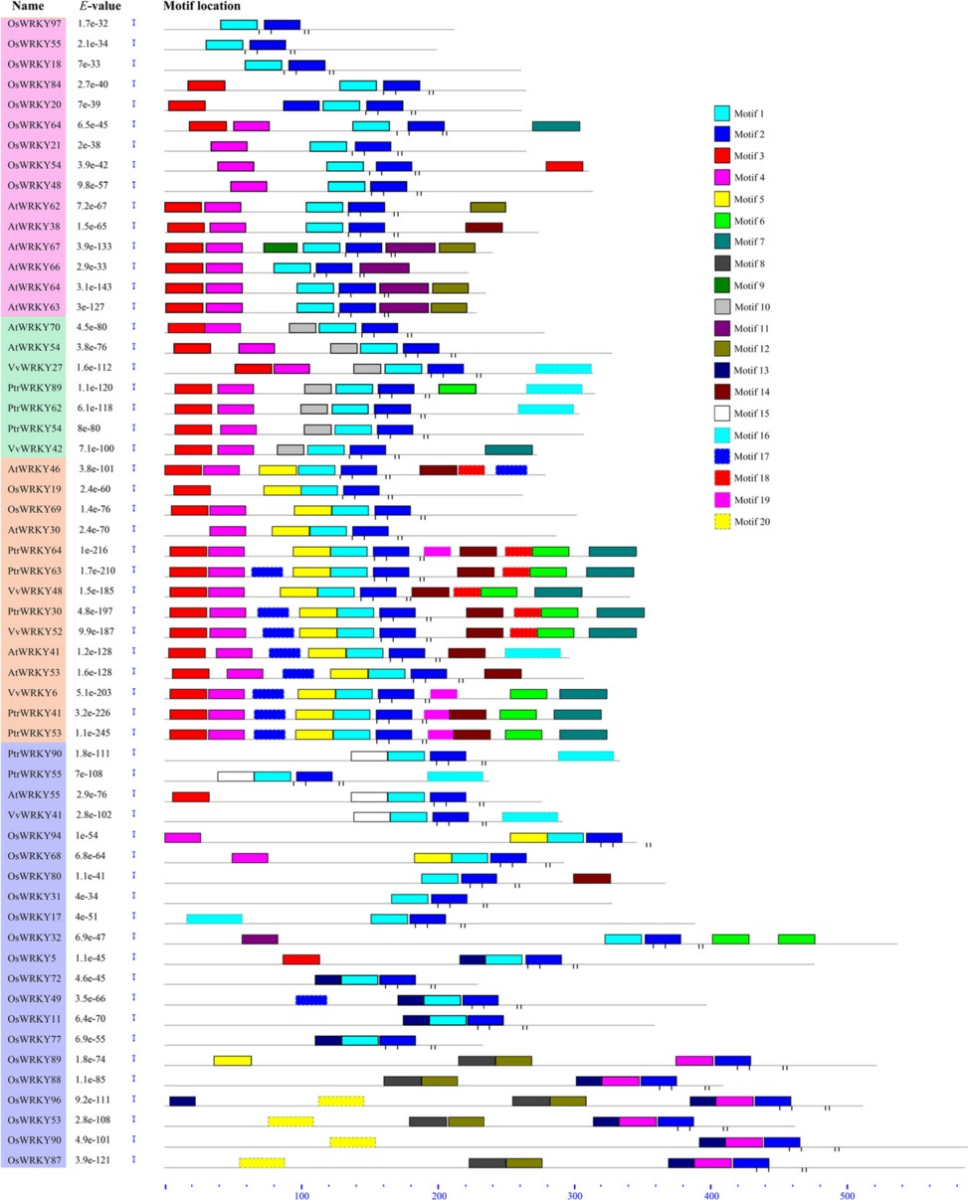
Distribution of conserved motifs in the WRKY III family members. All motifs were identified by MEME using the complete amino acid sequences of 57 *Populus*, grape, *Arabidopsis* and rice WRKY III proteins documented in Fig. 4. Names of all members among the defined gene clusters and combined *P-values* are shown on the left side of the figure, and motif sizes are indicated at the bottom of the figure. The positions of zn-finger domains predicted by the SMART tool. Database are indicated by vertical tick marks below each protein model. The different-colored boxes represent different motifs and their position in each WRKY III sequence. The length of protein can be estimated using the scale at the bottom. For details of the motifs see Additional file 1: Table S1

In addition to the WRKY exon/intron structure, other conserved motifs could be important to the diversified functions of WRKY proteins from rice, grape, *Arabidopsis* and *Populus* [21]. Therefore, we used the MEME web server to search the conserved motifs which were shared with the 57 WRKY proteins. A total of 20 distinct conserved motifs were found, and the conserved amino acid sequences and length of each motif are shown in Additional file 1: Table S1. Each of the putative motifs obtained from MEME was annotated by searching Pfam and SMART. Motif 1, motif 2 motif 4, motif 9 ane motif 12 were found to encode the WRKY DNA-binding domain, while the other motifs have not function annotation. As illustrated in Fig. 4, most WRKY members within the same clade, particularly the most closely related members, generally shared common motif compositions (e.g. *PtrWRKY27* and *VvWRKY52*), suggesting function similarities among WRKY proteins. Motif 2 is the most common motif, found in all fifty-seven WRKY III genes. Motif 9 was unique to the proteins in clade2 and other unique motifs (e.g. motif 17, motif 18 and motif 19) were found in clade 3; these motifs might be important to the functions of unique WRKY III protein. Motif 7 was mainly present in clade 3 except *OsWRKY64* and *VvWRKY42*, which existed in clade 1 and 2, respectively. To some extent, these specific motifs may contribute to the functional divergence of WRKY genes. The detailed information is shown in Additional file 1: Table S1. To predict the function of the different WRKY III genes, we searched the Gene Ontology (GO) Darabase [22], which provides a varity of functions for the 57 WRKY III protein sequences. This analysis predicted that all WRKY III genes contain some common functions, such as, sequence-specific DNA binding transcription factor activity, molecular function, regulation of transcription, biological process (Additional file 2: Table S2).

### Conserved microsynteny of WRKY III genes from poplar, grape, *Arabidopsis* and rice

**Fig. 5:**
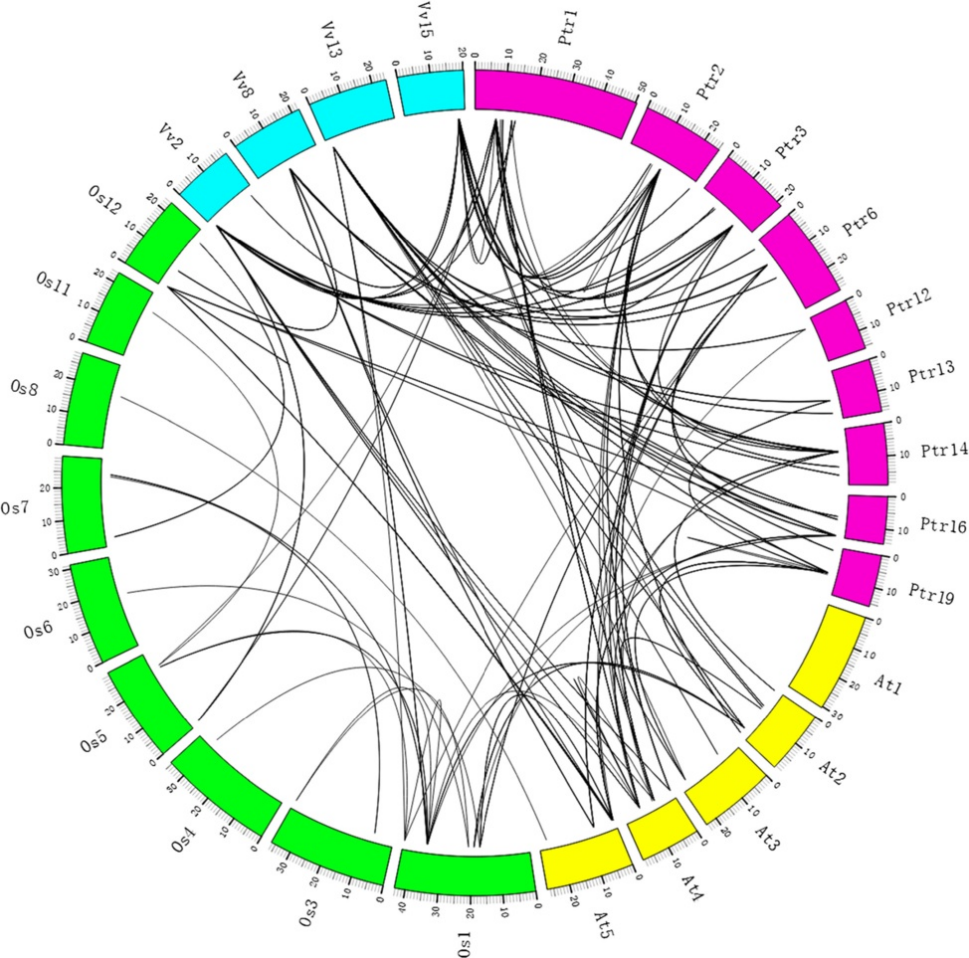
Extensive microsynteny of WRKY III regions across *Populus*, Grape, *Arabidopsis* and Rice. *Populus* chromosomes labeled Ptr, are indicated by rose red boxes. The Grape, *Arabidopsis* and Rice chromosomes, shown in different colors, are labeled Vv, At and Os, respectively. Numbers along each chromosome box indicate sequence lengths in megabases. The whole chromosomes of these four species, harboring WRKY regions, are shown in a circle. Black lines represent the syntenic relationships between WRKY regions

Microsynteny has been investigated across several plant species using whole-genome sequences to infer the location of homologous genes (orthology or paralogy) [23, 24]. To identify paralogous and orthologous relationships within the WRKY III genes, we performed microsynteny analysis of three dicotyledons (*Populus*, grape and *Arabidopsis*) and one monocotyledon (rice) to clarify the relationship of the WRKY genes between eudicots and monocots. The WRKY III genes of the four species were used as anchor genes to analyze the molecular history of the regions in which they resided. Through pairwise comparisons of flanking genes in the chromosomal regions containing WRKY III genes, there were three or more pairs among this area, which were considered conserved microsynteny (Fig. 5).

**Fig. 6:**
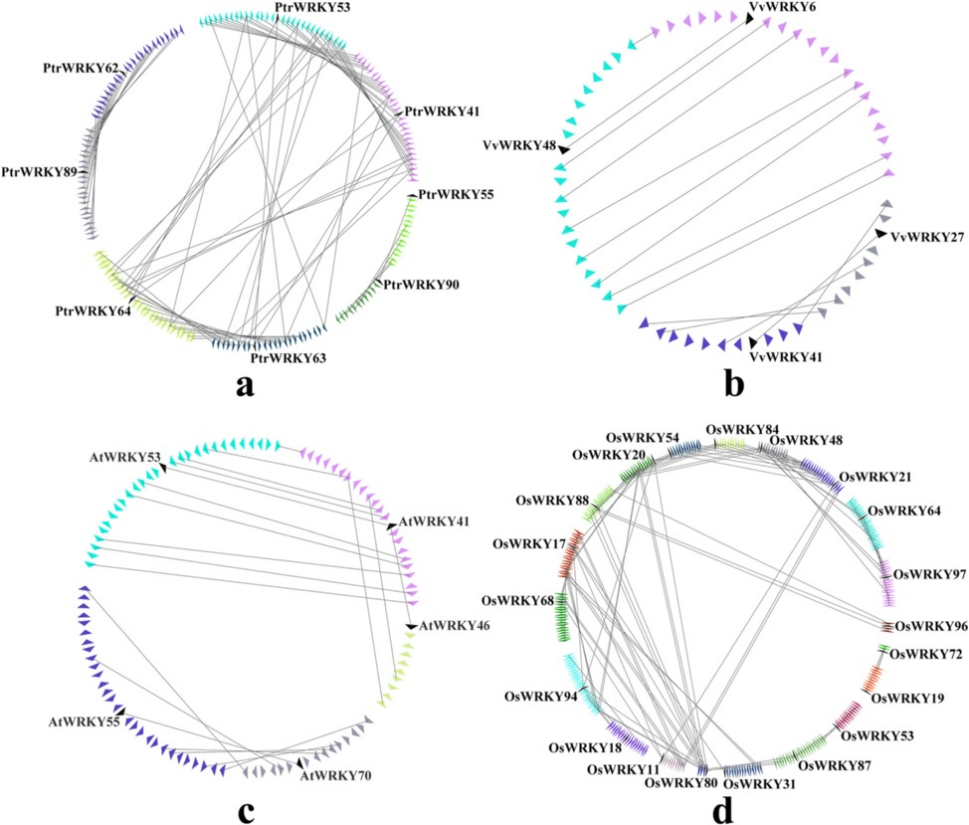
Microsynteny related to WRKY III families in (**a**) *Populus*; (**b**) grape; (**c**) *Arabidopsis*; (**d**) rice. **a**, **b**, **c**, **d**: The genomic fragments are represented by a series of triangles that represent a gene in a family and its flanking genes. The genes in the same fragment show the same color, except the gene in a family that is shaded by a black triangle. The triangle also indicates the gene’s orientation. A gray line connects the homologous genes on two fragments

Firstly, we analyzed the relationship of the WRKY III genes within each intraspecies, and identified 27 collinear gene pairs in the rice genome, a total of 8 collinear gene pairs in *Populus* genome, while only 3 and 2 collinear gene pairs in *Arabidopsis* and grape genome, respectively (Fig. 6, Additional file 3: Table S3a-d), which might have resulted from ancient processes during the course of evolution. In addition, 20 WRKY genes were not present in any microsynteny, for example, *PtrWRKY-30* and *54*, suggesting that there were independent duplication events except to the whole-genome duplication event.

**Fig. 7:**
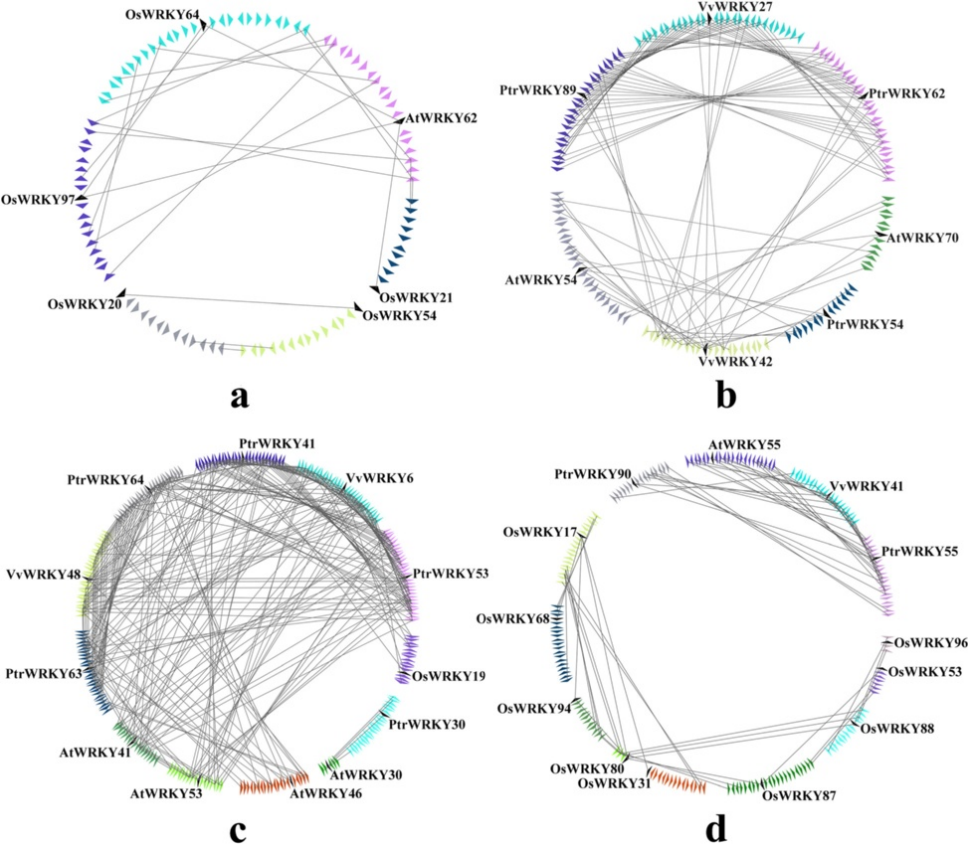
Microsynteny related to WRKY III families in (**a**) clade 1; (**b**) clade 2; (**c**) clade 3; (**d**) clade 4. **a**, **b**, **c**, **d**: The genomic fragments are represented by a series of triangles that represent a gene in a family and its flanking genes. The genes in the same fragment show the same color, except the gene in a family that is shaded by a black triangle. The triangle indicates the gene’s orientations. A gray line connects the homologous genes on two fragments

Subsequently, the corresponding interspecies microsynteny was also analyzed. Eighteen WRKY III genes were not detected in the interspecies microsynteny analysis, including *VvWRKY52*, five *AtWRKYIII*s, and twelve *OsWRKYIII*s. The map revealed 39 conserved syntenic segments distributed across different clades based on the phylogenetic tree analysis. A total of 15 orthologous gene pairs between *Populus* and grape were found, and 12 orthologous gene pairs between *Populus* and *Arabidopsis*, while we identified only one orthologous gene pair between *Populus* and rice (Fig. 7, Additional file 3: Table S3e-f), probably due to the closer relationship between *Populus* and grape/*Arabidopsis* versus *Populus* and rice. Insterestingly, some collinear gene pairs identified between *Populus* and grape/*Arabidopsis* were not identified between *Populus* and rice, such as *PtrWRKY54*/*VvWRKY42*, *PtrWRKY63/AtWRKY46*, which indicated that these orthologous pairs formed after rice diverged from the common ancestor of *Populus* and grape/*Arabidopsis*. Additionally, we observed a series of several-for-one microsyntenies between *Populus* and grape/*Arabidopsis* WRKY genes, while only *PtrWRKY30* and *AtWRKY30* exhibited one-for-one microsynteny and had no detected linkage with other WRKY genes, guessed these genes may have played a vital role in the expansion of the WRKY III gene family during evolution. For example, *PtrWRKY62*/*VvWRKY27*, *PtrWRKY62*/*VvWRKY42*, *PtrWRKY89*/*VvWRKY27*, *PtrWRKY89*/*VvWRKY42*, *PtrWRKY54/AtWRKY54*, *PtrWRKY54/ VvWRKY42*.

To estimate the extent of conserved gene content and order, the quality of the synteny was calculated [25]. The average synteny quality of the WRKY III genes from the three dicotyledons and one monocotyledon genomes was 25.85 %. The highest syntenic quality values were obtained between *Populus* and grape (42.19 %). Lower syntenic quality values were obtained between rice and *Populus* (14.29 %) and grape (17.78 %). The average synteny quality in the *Arabidopsis*/*Populus* and *Arabidopsis*/rice syntenic regions was 25.83 and 24.56 %, respectively, which was substantially lower than the 30.43 % observed in the *Arabidopsis*/grape synteny blocks. Details of this comparative analysis are shown in Table 2.

**Table 2:** Average relative syntenic quality of WRKY III genes in *Populus*, grape, *Arabidopsis* and rice

	Clade 1	Clade 2	Clade 3	Clade 4	Average
At-Os	24.56 %				24.56 %
At-Ptr		15.38 %	24.01 %	38.10 %	25.83 %
At-Vv		26.21 %	26.99 %	38.10 %	30.43 %
Os-Ptr			14.29 %		14.29 %
Os-Vv			17.78 %		17.78 %
Ptr-Vv		44.63 %	45.53 %	36.39 %	42.19 %
					25.85 %

### Gene duplication of WRKY III genes

The WRKY III gene family may have undergone many processes, including gene duplication resulting from large-scale duplication events (whole-genome or segmental duplication), or tandem duplication. Gene duplication has always been seen as an important source of, and contributor to, biological evolution. To better understand how WRKY III genes evolved, we investigated gene duplication events of the WRKY III family in *Populus*, grape, *Arabidopsis* and rice.

First, we analyzed the adjacent genes to determine whether tandem duplication has taken place. Paralogs were deemed to be tandem duplicated genes if two genes were separated by five or fewer genes in a 100-kb region on a chromosome. According to this, we observed that two places contain tandemly clustered genes: one tandem duplicated gene pair (*AtWRKY54* and −*90*, within an approximately 13.689-kb region) occurring in chromosome 1 of *Arabidopsis*, and the other tandem duplication gene pair (*OsWRKY48* and −*54*, within an approximately 20.112-kb region) in chromosome 5 of rice, and no pair was found to have been generated by tandem duplication in poplar and grape, suggesting that tandem duplication may have made little or no contribution to the expansion of the WRKY III gene family in these four species. Thus, we speculated that large-scale duplication events may have played an important role in the evolution of the WRKY III family genes in *Populus*, grape, *Arabidopsis* and rice.

To investigate this possibility, we analyzed the gene similarity in the WRKY III flanking regions. If five or more protein-coding gene pairs flanking the anchor point were ligatured with the best non-self match (E-value <1e-10), we considered these gene pairs to be conserved and defined these two regions as derived from a large-scale duplication event.

Consequently, significant collinearity may exist in the WRKY III regions. In poplar, we identified five conserved genes flanking three pairs, *PtrWRKY41/-64*, *PtrWRKY41/-63* and *PtrWRKY90/-55*. Five other pairs of WRKY genes (*PtrWRKY63/-53*, *PtrWRKY64/-53*, *PtrWRKY64/-63*, *PtrWRKY62/-89* and *PtrWRKY41/-53*) contained more than five pairs of conserved flanking genes. Therefore, these gene pairs are thought to have been created by large-scale duplication. In grape, genes flanking both pairs (*VvWRKY6/-48* and *VvWRKY41/-27*) were found to be conserved. In *Arabidopsis*, the relationships between three duplicated gene pairs were judged, *AtWRKY41/-53*, *AtWRKY55/-70* and *AtWRKY46/-41*. In rice, 20 out of 28 WRKY III genes (approximately 78.57 %) were present in duplicated chromosomal regions. Four gene pairs (*OsWRKY97/-64*, *OsWRKY21/-48*, *OsWRKY21/-84* and *OsWRKY21/-54*) were found to have been involved in large-scale duplication.

### Strong purifying selection for WRKY III genes in *Populus*

The results showed in the previous section suggested that almost the entire WRKY III gene family of *Populus* was expanded by large-scale gene duplication. To better understand the evolutionary constraints acting on this gene family, we calculated the Ka/Ks ratios for eight unambiguous pairs of WRKY III paralogs in the network of duplicated regions of *Populus*. Generally, a Ka/Ks < 1 indicates the functional constraint with negative or purifying selection of the genes, a Ka/Ks ratio of 1 means that the genes are drifting neutrally, and Ka/Ks > 1 indicates accelerated evolution with positive selection.

**Fig. 8:**
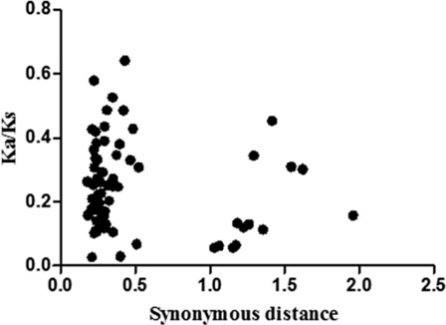
Scatter plots of the Ka/Ks ratios of duplicated WRKY III genes in *Populus*. The Y- and X-axes denote the Ka/Ks ratio and synonymous distance for each pair, respectively

Assuming that synonymous silent substitutions per site (Ks) occur at a constant rate over time, we can use the conserved flanking protein-coding genes to estimate the dates of the large-scale duplication events; the pairwise Ka/Ks ratios were also calculated for the duplicated non-WRKY III genes (flanking genes) between the duplicated regions containing *WRKYIIIs* in *Populus*. We discarded any Ks values >2.0 because of the risk of saturation [26, 23]. All the Ka/Ks ratios from the eight poplar WRKY paralogous pairs were less than 0.4 (Table 3). Based on this analysis, we concluded that the WRKY III gene family had mainly been subjected to strong purifying selection and that the WRKY III genes are slowly evolving at the protein level. Interestingly, all the Ka/Ks values for the 82 pairs of duplicated non-WRKY III genes were lower than 1 (Fig. 8), clearly indicating that these genes are evolving under purifying selection.

**Table 3:** Estimates of the dates for the large scale duplication events in poplar

Duplicated Hsf gene pairs	Number of conserved flanking protein-coding genes	Ka/Ks (mean ± s.d.)	Ks (mean ± s.d.)	Date (mya)
PtrWRKY41/53	21	0.2956 ± 0.1619	0.2958 ± 0.0823	16.2512
PtrWRKY62/89	18	0.2260 ± 0.1143	0.3045 ± 0.0941	16.7297
PtrWRKY64/63	15	0.3577 ± 0.1307	0.2857 ± 0.0777	15.6974
PtrWRKY64/53	7	0.2751 ± 0.1394	1.0270 ± 0.6001	56.4264
PtrWRKY63/53	6	0.2071 ± 0.1755	1.1829 ± 0.7230	64.9923
PtrWRKY41/64	5	0.2130 ± 0.1245	1.2803 ± 0.3083	70.3443
PtrWRKY41/63	5	0.2540 ± 0.0519	1.2109 ± 0.0761	66.5330
PtrWRKY90/55	5	0.3287 ± 0.0633	0.2635 ± 0.0936	14.4753

The approximate date of the duplication event was calculated using the mean Ks and an estimated divergence rate of 9.1 × 10^−9^ synonymous mutations per synonymous site per year, as previously proposed for *Populus*. The eight duplication blocks were estimated to have occurred between 14.48 to 70.34 Mya (Table 3). We concluded that the large-scale duplication events involving *Populus WRKYIIIs* all occurred within the last 14.48–70.34million years.

**Fig. 9:**
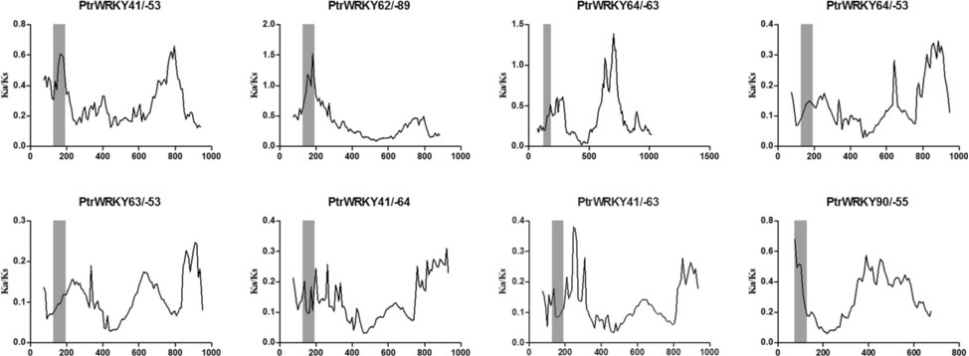
Sliding window plots of representative duplicated WRKY III genes in *Populus*. As shown in the key, the gray blocks indicate the positions of the WRKY domain. The window size was 150 bp, and the step size was 9 bp

During positive selection, a few individual codon sites could be masked by overall strong purifying selection; therefore, we performed a sliding-window analysis of Ka/Ks ratios between each pair of WRKY III paralogs, which were derived from gene duplication events in *Populus* (Fig. 9). As predicted from the basic Ka/Ks analysis, the sliding window analysis clearly showed that numerous sites/regions are under neutral to strong negative or purifying selection. Using this analysis, the majority of Ka/Ks ratios across coding regions were far below one, but one or a few distinct peaks (Ka/Ks >1) were shown in Fig. 9. Consistent with functional constraints being dominant in these domains, the domains of more than half of *WRKYIII*s generally had lower Ka/Ks ratios than the regions outside of them (peaks). Moreover, the conserved domains of *WRKYIIIs* stronger purifying selections, with Ka/Ks ratios < 1. One exception (*PtrWRKY62* and −*89*) revealed sites with higher Ka/Ks ratios (Ka/Ks ratios >1) in their domains, indicating positive selection in this region, and implying these two genes experienced somewhat different selective pressure, which reveals the domains showing a higher evolutionary rate that is otherwise hidden in the average value of the Ka/Ks ratio. In addition, positive selection contributes to a higher Ka/Ks ratio, yet it does not guarantee that the gene-average Ka/Ks ratio is over one.

Combining Ka/Ks ratios and a sliding-window analysis, we provided evidence suggesting that negative or purifying selection might have played an important role in the evolution of the WRKY III gene family in *Populus.*

### Expression patterns of *Populus* WRKY III genes in various tissues

**Fig. 10:**
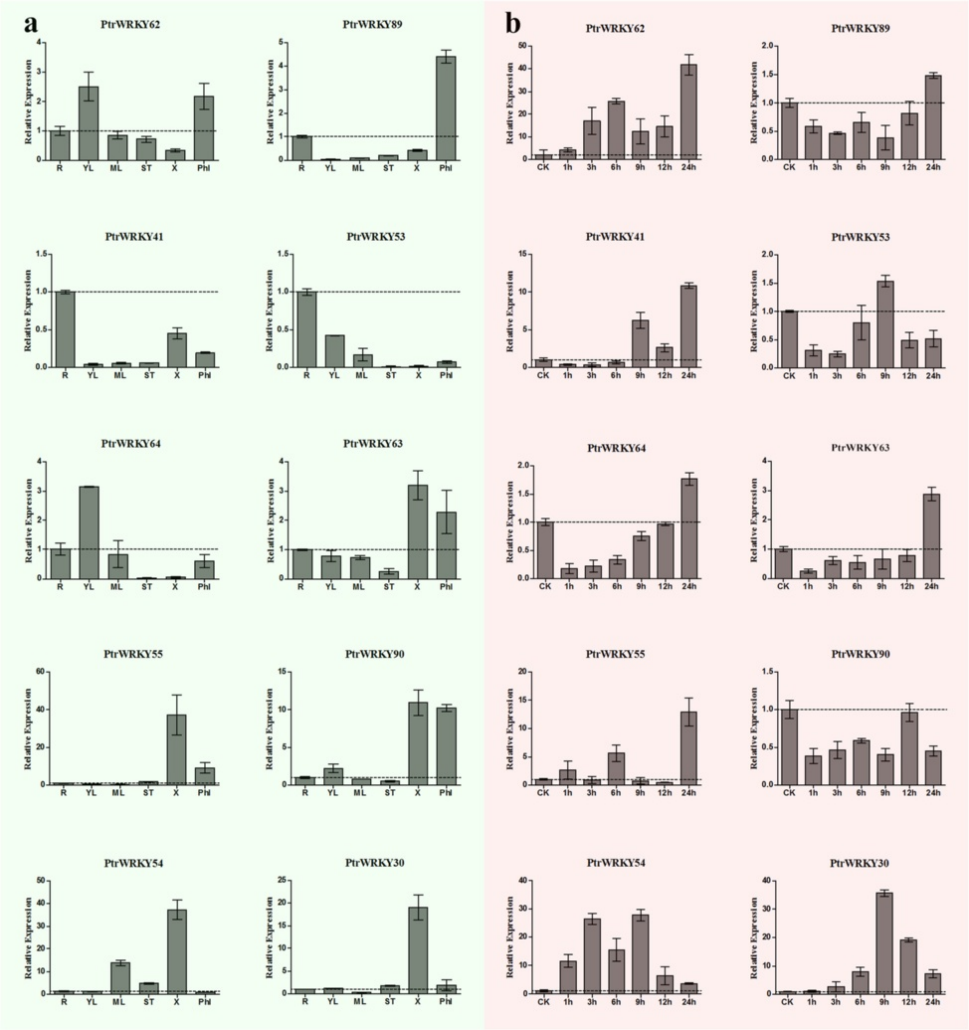
qRT-PCR expression levels of selected *PtrWRKY* genes following SA (100uM), and different tissues. The Y-axis indicates the relative expression levels; 0, 1, 3, 6, 9, 12, and 24 (X-axis) indicate hours of treatment. Mean values and standard deviations (SDs) were obtained from three biological and three technical replicates. **a** Expression patterns of WRKY III genes from *Populus* in various tissues. R, roots; YL, young leaves; ML, mature leaves; ST, stems; X, xylem; Phl, phloem. **b** Expression levels of selected *PtrWRKY* genes under SA treatment. Horizontal discontinuous lines marks the 1.0 value

To gain an insight into the potential functions of *Populus* WRKY III genes during development, we used qRT-PCR to determine the expression patterns of 10 *PtrWRKY* genes in six organs/tissues (roots, young leaves, mature leaves, stems, xylem and phloem). The 10 *Populus* WRKY genes showed significantly different tissue-specific expression patterns in the different tissues (Fig. 10a). Among the 10 *Populus* WRKY genes, two showed the highest transcript accumulation in the roots (*PtrWRKY41* and −*53*), two in young leaves (*PtrWRKY62* and −*64*), one in the phloem (*PtrWRKY89*) and five in the xylem (*PtrWRKY30, −54, −55, −63 and −90*). Most of the paralogous pairs had similar expression patterns; for example *PtrWRKY41/-53* and *PtrWRKY55/-90*, which are highly expressed in roots and xylem, respectively, with little or no expression in other tissues. Nevertheless, some of the paralogous pairs showed different expression patterns; for example, *PtrWRKY64* is expressed at a high level in young leaves, while its paralog, *PtrWRKY63,* is highly expressed in the xylem.

### Expression profiles of *Populus* WRKY III genes in response to different stress treatments

Environmental stress can affect a plant’s health and growth, and influence the regulation of important genes. Under adverse conditions, many stress-related genes are induced to help plants deal with stress. Therefore, it is necessary to identify the master regulators of stress responses in *Populus*, as well as their regulatory pathways. To explore the stress responses involving the *Populus* WRKY III genes, we used qRT-PCR to analyze their expressions in response to different treatments. The results of SA treatment showed a wide variety of *PtrWRKYIII* gene expression profiles (Fig. 10b). A total of 9 genes were up-regulated by SA treatment, *PtrWRKY90* was obviously down-regulated at all time points. Among these genes, the highest expression levels of *PtrWRKY54*, −*30* and −*53* occurred 9 h after treatment: *PtrWRKY54* and −*30* were strongly up-regulated (by more than 28-fold and 36-fold, respectively). The expressions of six genes (*PtrWRKY89, −62, −64, −63, −41* and −*55*) peaked at the last time point (24 h); *PtrWRKY41* and −*55* were up-regulated by more than 11-fold and *PtrWRKY62* showed the greatest upregulation (by more than 42-fold. In addition, a few paralogous pairs shared similar expression profiles. For instance, *PtrWRKY64* and −*63* showed were both up-regulated at 3 h, with their highest levels at 24 h, in response to SA treatment. *PtrWRKY89* and −*62* had the same trend after 3 h, with high expression at 24 h. Different expression patterns between two paralogous genes were also observed. For example, the highest expression level of *PtrWRKY41*was observed at 24 h after SA treatment (by more than 10-fold), while that of *PtrWRKY53* was up-regulated by 1.5-fold at 9 h.

**Fig. 11:**
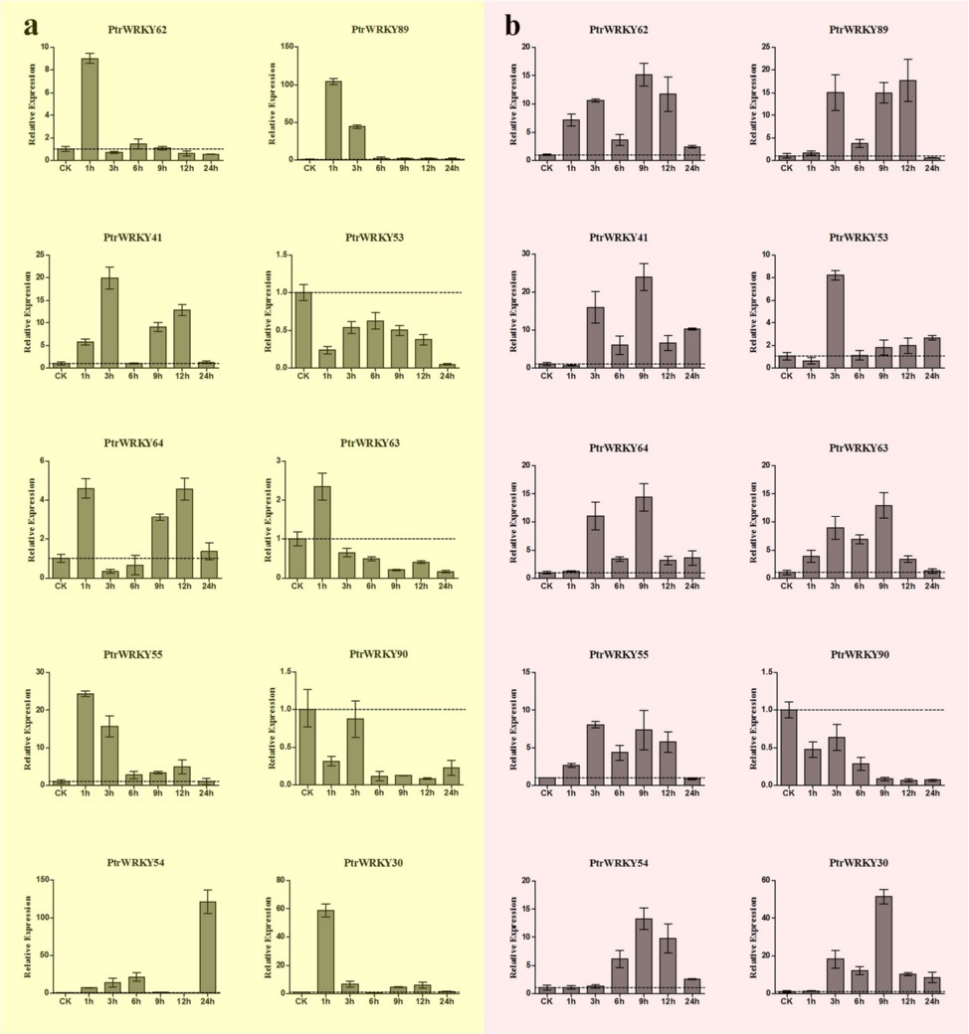
qRT-PCR expression levels of selected *PtrWRKY* genes following PEG-6000 (25 %) treatment, and ABA (100uM) treatments. The Y-axis indicates the relative expression levels; 0, 1, 3, 6, 9, 12, and 24 (X-axis) indicate hours of treatment. Mean values and standard deviations (SDs) were obtained from three biological and three technical replicates. **a** Expression levels of selected *PtrWRKY* genes under PEG treatment. **b** Expression levels of selected *PtrWRKY* genes under ABA treatment. Horizontal discontinuous lines marks the 1.0 value

We investigated the expression patterns of *Populus* WRKY III genes under drought stress: the leaves were sprayed with 25 % PEG and ABA solution, respectively, to imitate drought treatment. Significant expression level changes were observed for 10 *PtrWRKYIIIs* under the two treatments, of which 8 were up-regulated by PEG treatment, 9 were up-regulated by ABA treatment, however, *PtrWRKY90* was down-regulated at different time-points following the two treatments (Fig. 11). It suggested that more 80 % of the *PtrWRKYIIIs* analyzed were drought responsive. Examination of the number of *PtrWRKYIIIs* with significant expression level changed at different time-points of treatment showed that the expression of 6, 1 and 1 *PtrWRKYIIIs* were changed after PEG treatment for 24, 3 and 1 h, respectively, and the expression of 6 and 3 *PtrWRKYIIIs* were changed after ABA treatment for 9 and 3 h, respectively (Fig. 11). It suggested that the majority of *PtrWRKYIIIs* have altered expression levels at the time-point of 1 h and 9 h under PEG and ABA treatment. Under PEG and ABA trements, only *PtrWRKY90* was down-regulated at all time points, which indicated that these genes may play different roles in the response to different drought stresses.

## Discussion

The WRKY transcription factor gene family is involved in the regulation of a variety of processes. In the present study, the complex features and functions of this group of proteins have been studied in the model herbaceous plant *Arabidopsis*, in rice, in the woody plant poplar and in grape.

There are anatomical and physiological differences between the four species, which might be reflected in the diversity of WRKY III genes’ structure and conserved motifs. Exon-intron structural diversification plays an important role in the evolution of many gene families, and exon-intron gain or loss may be caused by the rearrangement and fusions of different chromosome fragments. We identified that the 57 WRKY III genes contain different numbers of exons, indicating that there is some diversity in these four species. For example, the WRKY gene *VvWRKY48* has five exons, while other genes in the same phylogenetic clade (clade 3) have three exons. Nevertheless, the characteristics of exon/intron structure and motif composition were relatively conserved in recent paralogs: most closely related genes within the same clade shared similar gene structures, either in their intron numbers or exon lengths. The MEME server identified the different conserved protein motifs that are present in each of the WRKY proteins. Some closely related members shared similar structures, implying functional similarities for these WRKY proteins. The specific sequence motifs present in each clade may impart specific functions to the WRKY proteins. The similarities in gene structure and motif composition of most WRKY proteins consistented with phylogenetic analysis of the WRKY III gene family. The differences in these characteristics among the different clades suggested that the WRKY members were functionally diversified.

To explore how the WRKY III gene family evolved, we performed a genome-wide comparison of plant WRKY members from monocots (rice) and eudicots (*Populus*, grape and *Arabidopsis*). Considerable phylogenetic analysis of WRKY proteins has been conducted in poplar, grape, rice and *Arabidopsis*, respectively. To obtain an overall picture of the 57 WRKY III proteins and their relationships with each other, a phylogenetic tree of WRKY III proteins was constructed, which divided the 57 WRKY members into four clades. The plant WRKY III members from eudicots (*Arabidopsis*, grape and poplar) appear to be more closely related to each other than to WRKY III genes of the monocots (rice). The presence of four distinct clades of WRKY III genes and the presence of both monocots and eudicots members in all four clades indicated that WRKY III genes diversified before the monocot-eudicot split (Fig. 2). In addition, these clades include 19 pairs of homologous genes (17 pairs of paralogous genes and two pairs of orthologous genes), two of the orthologous genes pairs from dicotyledons (poplar and grape), which was consistent with the fact that both poplar and grape are eurosid I members and therefore more closely related than *Arabidopsis*, which belong is a eurosid II member. However, seven pairs were genetically linked to each other on their corresponding chromosomal locations, which indicated that there were very few tandem duplications among the WRKY III genes. According to the criterion for tandem duplication, two pairs of orthologous genes were deemed to be tandem duplicated genes, which indicated that tandem duplication has made little contribution to the expansion of the WRKY III gene family.

Gene duplication is a major evolutionary mechanism for generating novel genes, which helps organisms adapt to different environments. Tandem and large-scale duplications (whole-genome or segmental duplication) are well-known patterns of gene duplication in plants [23]. In our analysis, we found that a high proportion of WRKY III genes are distributed in duplicated blocks, suggesting that large-scale duplication contributed significantly to the expansion of the WRKY III gene family.

During evolution, eukaryotic genomes have retained genes on corresponding chromosomes (synteny) and in corresponding orders (collinearity) to various degrees. Synteny broadly refers to parallels in gene arrangement in dissimilar genomes. Microsynteny has been previously described among many monocot and eudicot species [27]. In our study, according to the microsynteny analysis, no microsynteny relationships among *VvWRKY52*, *AtWRKY-30, 38, 63, 64, 66* and *67*, *OsWRKY-77, 32, 55, 68, 5, 84, 49, 89, 69, 90* and *18* with other WRKY III members in these three dicot (*Populus*, grape and *Arabidopsis*, respectively) and one monocot (rice) genomes were observed, indicating that either these genes are ancient genes without detectable linkage to other WRKY III genes or that they were formed through complete transposition and loss of their primogenitors. In the four WRKY III clades, genes from poplar, grape, *Arabidopsis* and rice exhibited high levels of microsynteny, which indicated that the WRKY III genes existed before the divergence of the four genomes (poplar, grape, *Arabidopsis* and rice). Several previous studies have shown that WRKY III domain genes have been duplicated independently after the divergence of monocots and dicots (160 Mya) [2, 28, 19]. Ling et al. [9] reported that in cucumber (*Cucumis sativus*) *CsWRKY* family, a divergence generated in the number of group-III WRKY genes resulted from different types of duplication events that occurred after the divergence of the eurosids groups I and II (110 Mya) [9]. In the current study, a large amount of microsynteny was detected in the three dicotyledons (poplar, grape and *Arabidopsis*), and little or no microsynteny between monocotyledon (rice) and the three dicotyledons, which was consistent with the evolutionary relationships between monocot and eudicot species. The low (25.85 %) synteny quality of WRKY III genes from monocotyledon (rice) and three dicotyledons (poplar, grape and *Arabidopsis*) may have been due to the fact that these plants are not closely related; moreover, the gene density differed between rice and the three other eudicot species. Significantly, the number of synteny blocks (27) within the rice genome is much higher than the number of synteny blocks of the three other eudicot species genomes, which suggested that rice WRKY III genes may have undergone large-scale duplication events and less subsequent rearrangement (Fig. 6 and Additional file 3: Table S3). In three eudicot species, the number of synteny blocks (8) within the *Populus* genome is much more than that (2 or 3) between grape or *Arabidopsis* genomes, which may reflect the fact that *Populus* WRKY III genes have undergone large-scale duplication events. Thus, our results indicated that one important factor in the expansion of WRKY III genes was the occurrence of large-scale duplication events.

The nature of internal microsynteny in the four species provided further evidence that a large-scale duplication predated speciation. Assuming genome duplication preceded speciation, the microsynteny map should exhibit paired microsynteny blocks, each corresponding to the offspring of the ancient duplication event and each exhibiting comparable levels of microsynteny between the four species. In addition, if a single large-scale duplication event generated the homologous segments, they should all have been created at the same time.

*Populus*, as a large and long-lived woody plant, had a different life history compared with *Arabidopsis*, grape and rice, and is likely to be more complex with respect to development and gene regulation networks. WRKY genes were found to be expressed in many tissues and seem to be involved in regulating plant developmental and physiological processes. There was considerable evidence that WRKY genes play crucial roles in the responses to abiotic and biotic stress-induced defense signaling pathways [29]. From an applied perspective, the identification of WRKY III genes with potential value in different tissues and in the stress resistance of *Populus* might be followed by targeting such genes to improve abiotic and biotic stress responses.

The qRT-PCR expression profiles generated in this study (Figs. 10 and 11) revealed WRKY III protein genes have a broad expression pattern across different tissues and/or organs in poplar and different expression patterns for each *PtrWRKY* gene under specific treatments. This data provided a useful resource for future gene expression and functional analyses. Among the 10 poplar genes, half of them (*PtrWRKY30, −55, −63, −90, −54*) exhibited high expression levels in the xylem, suggesting their importance during xylem formation. Three genes (*PtrWRKY64, −41, −53*) were preferentially expressed in young leaves and roots, which could indicate that these genes play significant roles in leaf and root expansion. This conclusion is very important for future research on drought resistance of poplar. Some of the paralogous gene pairs showed similar expression patterns (*PtrWRKY41/-53* and *PtrWRKY55/-90*), suggesting that these genes have not diverged substantially after duplication, and have retained redundant functions in regulating tissue development.

In plants, many stress-related genes are induced in response to adverse conditions. For instance, expression of *GsWRKY20* in *Arabidopsis* enhances drought tolerance and regulates ABA signaling [30]. Overexpression of *WRKY25* or *WRKY33* was sufficient to increase *Arabidopsis* NaCl tolerance and increase sensitivity to ABA [14]. In *Populus*, 10 WRKY genes, belonging to group III were induced by varieties of stresses, such as cold, salinity, SA and drought, but no further analysis was performed [6]. Therefore, in this work, we performed qRT-PCR of *PtrWRKYIII* genes with SA treatment and drought (ABA /PEG) treatment to detect whether these genes are related to defense against disease and drought. The qRT-PCR results showed that most of the genes were up-regulated by the three treatments, with the exception of *PtrWRKY90*. The result was a little different from those found in poplar by Hongsheng He et al. This difference may be explained by the following: first, the growth conditions were different. The materials we sampled were using six-week-old seedlings and growing in tissue culture vessels under aseptic conditions, while the other plants were used six-month-old seedlings which grown in a greenhouse. Second, the leaves we used were young leaves, while the other experiments all sampled mature leaves, ect. The same gene showed different expression patterns under different stresses; e.g., *PtrWRKY89* was strongly induced by ABA and PEG treatment, with expression increased by more than 18-fold and 104-fold, respectively; however, the relative expression was only 1.5-fold higher under SA treatment. This result was consistent with the previous performed studies [6]. Interestingly, *PtrWRKY89* was reported to play a regulatory role in the SA signaling pathway to increase poplar’s defense [7]. This gene was suggested to be involved in both disease and drought resistance. Thus, the drought resistance function of *PtrWRKY89* requires further research. Grape *WRKY27* were significantly induced by drought and SA treatments, suggestting that *VvWRKY27* play a role in mediating plant defense response [31]. WRKY transcription factors have been identified as key components in the ABA signaling pathways [32, 33]; in grape, *VvWRKY27* may participate in an ABA-dependent signal pathway [34]. Base on the microsyteny analysis, we auspiciously found there exsited highly conserved microsynteny relationship between *VvWRKY27* and *PtrWRKY89/-62*. And combined with phylogenetic analysis, we speculated poplar WRKY III members might have the similar biological function with *VvWRKY27* in defense to virious responses. Some genes have a variety of functions, for example, *PtrWRKY30* accumulated the highest level transcripts approaching 35-fold, 58-fold and 51-fold, by SA, PEG and ABA treatment, respectively.

Duplicated genes face three outcomes: non-functionalization (one copy becomes silenced); neo-functionalization (one copy acquires a novel, beneficial function, while the other copy retains the original function) or sub-functionalization (both copies become partially compromised by the accumulation of mutations) [35, 36, 21]. Paralogs originating from duplication within one organism may have more divergent functions. In *Arabidopsis*, *AtWRKY41* and *AtWRKY53* are paralogoues but have different expression patters, *AtWRKY53* was more sensitive to SA treatment than papalogue *AtWRKY41* [37]. In our study, we noticed that *PtrWRKY41* and *PtrWRKY53* and different expression trajectories by the SA treatment, *PtrWRKY41* was more sensitive than papalogue *PtrWRKY53*, which was consistent with the previous study. Throughout evolution, the plants diversified and acquired new genes that may have important roles in plant development. Several pairs of paralogs have different expression patterns, suggesting that they play diverse roles in Populus development. For example, *PtrWRKY55/PtrWRKY90* are mainly expressed in the xylem and phloem. Upon SA treatment, *PtrWRKY55* was highly expressed at 24 h, while its paralogs gene *PtrWRKY90* was down-regulated at all time points. Several pairs of paralogs showed similar expression, which suggests that they may share a common or similar function. For example, *PtrWRKY63/PtrWRKY64* expression peaked at 24 h, 1 h, and 9 h in response to SA, PEG, and ABA, respectively, indicating that the responses of paralogs to stress conditions did not undergo much divergence during the evolution of each gene after duplication and that the duplicated genes may have redundant functions in response to drought stress and upon treatment with signaling substances such as salicylic acid (SA).

## Conclusions

In the current study, these 57 members of the WRKY III were analyzed, a comprehensive analysis including their chromosomal location, phylogeny, gene structure, conversed motifs, conserved microsynteny and gene duplication, and the expression profiling of 10 WRKY III genes in poplar was performed. These WRKY III genes were clustered into four clades based on phylogenetic analysis. In each clade, the characteristics of exon/intron structure and motif compositions were relatively conserved. Although the genomes sequence of the four species has been reported, the comprehensive analysis of WRKY III genes and funtional studies on poplar genes are still lag behind. Comparisons among the WRKY III genes across the four species genomic sequences demonstrated extensive synteny plus the existence and timing of one or more large-scale genome duplications in the evolution. Our results indicated that the vast majority of WRKY III gene of *Populus* was expanded by large-scale gene duplication. These genes had mainly been subjected to strong purifying selection and slowly evolved at the protein level. Furthermore, the expression pattern of *PtrWRKYIII* gene identified that these genes play important roles in the xylem during poplar growth and development, and may play crucial role in defense to drought stress. Here, we speculate that *PtrWRKY* proteins play fundamental roles in various plant developmental processes. The systematic analysis of the WRKY III family genes and the preliminary results presented here may aid in the selection of appropriate candidate genes for further characterization of their biological functions in poplar.

## Methods

### Database searches for highly conserved WRKY III genes

The WRKY III genes of four species (*Populus trichocarpa*, *Arabidopsis*, rice and grape) were downloaded from the latest version of the Phytozome database (v9.1). Fifty-seven WRKY III genes (13 *AtWRKYs*, 6 *VvWRKYs*, 28 *OsWRKY*s and 10 *PtrWRKYs*) were identified. The accession numbers of published WRKY III genes from *Populus*, *Arabidopsis*, rice, and grape are listed in Table 1. WRKY III gene information, including the number of amino acids, ORF lengths and chromosome locations, was obtained from the Phytozome database. Physical parameters of the WRKY III proteins, including molecular mass (kDa), and isoelectric point (pI) were calculated using the compute pI/Mw tool in ExPASy (http://www.expasy.org/tools/), with parameters (resolution) set to ‘average’ [38].

### Chromosomal location

Genes were mapped onto each chromosome based on publicly available information about the chromosome locations provided in the Phytozome database (http://www.phytozome.net). Chromosomal location images of WRKYIII genes were subsequently generated using the MapInspect software (http://www.plantbreeding.wur.nl/uk/software_mapinspect.html).

### Phylogenetic analysis of Group III WRKY family

Multiple sequence alignments of the full-length protein sequences from *Populus*, *Arabidopsis*, rice and grape were performed using MEGA6.0 [39] with default parameters. A phylogenetic tree based on the alignment was constructed using MEGA6.0 and the Neighbor-Joining (NJ) method [40], and the Maximum Parsimony (MP) method [41] was also used to create a phylogenetic tree and to validate the results from the N-J method. Bootstrap analysis was performed using 1000 replicates in the pairwise gap deletion mode, which allows divergent domains to contribute to the topology of the NJ tree [42].

### Exon-intron structure and conserved motif analysis

The exon and intron structures of individual WRKY III genes were determined using the Gene Structure Display Server (GSDS; http://gsds.cbi.pku.edu.cn/) via alignment of the CDS with their corresponding genomic DNA sequences [43].

Conserved proteins motifs were analyzed Online MEME (Multiple Expectation Maximization for Motif Elicitation) [44]. The parameters were as followings: number of repetitions-any, with maximum number of motifs = 20, and the optimum motif width was constrained to between 6 and 200 residues. In addition, structural motif annotation was performed using the Pfam (http://pfam.sanger.ac.uk/search) and SMART (http://smart.embl-heidelberg.de/) tools. The WRKY III genes function annotation were achieved using the Gene Ontology (GO; www.geneontology.org).

### Microsynteny analysis and gene expansion patterns

Microsynteny analysis across the four species was performed based on comparisons of the specific regions containing WRKY III genes. Similarly, the WRKY genes of *Populus*, grape, *Arabidopsis* and rice were grouped according to their classification in the phylogenetic tree. MicroSyn was used to detect microsynteny [45]. Before starting the microsynteny analysis, three files were generated: the gene list file, the CDS file and the gene identifier file. The microsynteny diagram was achieved by loading these files. A syntenic block was defined as a region where three or more conserved homologs were located within 15 genes upstream and downstream between genomes. The relative syntenic quality in a region was calculated from the sum of the total number of genes in both conserved gene regions, excluding retroelements and transposons, and collapsing tandem duplications [25].

To better understand how WRKY III genes evolved, i.e., whether they arose from a large-scale duplication event (duplicated blocks derived from whole-genome or segmental duplication) or tandem duplication, we examined the physical locations of all WRKY III genes. To categorize the expansion of the WRKY III genes, tandem duplication was determined if two genes were separated by five or fewer genes in a 100-kb region on a chromosome [46]. Two regions were considered to have originated from a large-scale duplication event when five or more protein-coding gene pairs flanking the anchor point were ligatured with the best non-self match (*E-value* < 1e-10) [47, 36].

### Ks analysis of homologous segments

The duplicated gene pairs within each duplicated block or divergence of homologous segments were used to calculate the number of synonymous substitutions per synonymous site (Ks) and the Ka/Ks ratio, which is the ratio of the number of nonsynonymous substitutions per non-synonymous site (Ka) to Ks. Protein sequences of the gene pairs were first aligned using Clustal *X*2.0, then the multiple sequence alignments of proteins and the corresponding cDNA sequences were converted to codon alignments using PAL2NAL (http://www.bork.embl.de/pal2nal/) [48]. Finally, the resulting codon alignment was used to calculate Ks and Ka using the CODEML program of PAML [49]. A sliding window analysis of nonsynonymous substitutions per non-synonymous site Ka/Ks ratios was conducted with the following parameters: window size, 150 bp; step size, 9 bp [24].

When dating large-scale duplication events, Ks can be used as the proxy for time. For each pair of duplicated regions, the mean Ks of the flanking conserved genes were calculated, and these values were then translated into divergence time in millions of years, assuming a rate of 9.1 × 10^−9^ substitutions per site per year. The divergence time (T) was calculated as T = Ks / (2 × 9.1 × 10^−9^) × 10^−6^ million years ago (Mya) [21].

### Plant materials, growth conditions, and stress treatments

Asexually reproduced six-week-old *Populus deltoides* cv. ‘Nanlin95’ seedlings that were grown in a tissue culture laboratory under long day conditions (14-h light from 08:00 to 22:00) at 25–27 °C were used to assay gene expression levels in all experiments. Rooted seedlings of about 10 cm in height were selected for stress treatments. For the stress treatments, young leaves were sprayed with either 25 % polyethylene glycerol-6000 (PEG) or 100 μM abscisic acid (ABA) or 100 μM salicylic acid (SA) solution and sampled at five time points (1,3, 6, 9, 12, and 24 h) after treatment. Untreated seedlings were used as controls. After all of the materials were collected, the samples were immediately frozen in liquid nitrogen and stored at −80 °C until RNA extraction. Three biological and three technical replicates were employed per sample.

### RNA extraction and quantitative real-time reverse transcription PCR (qRT-PCR) analysis

Total RNA samples were extracted from leaves and stem tips using the Trizol reagent. Total RNA samples were extracted from root, xylem and phloem using an optimized, modified Cetyl trimethylammonium bromide procedure. The first-strand cDNA was synthesized using a PrimeScript™ RT Reagent Kit (TaKaRa) according to the manufacturer’s instructions. Gene-specific primers were designed and checked for specificity using Primer Premier 5.0 (Additional file 4: Table S4) and the NCBI primer Blast tool, respectively. In this study, the poplar housekeeping ubiquitin gene (UBQ, gene ID: Potri.001G418500) was used as reference for normalization because of its stable expression pattern [50]. qRT-PCR was performed in a 20 μl volume, which contained 10 μl of 2× SYBR® Premix Ex Taq™ (TaKaRa, Japan), 0.4 μl of 50× ROX Reference Dye, 2 μl diluted cDNA template, 0.8 μl of each specific primer, and 6 μl ddH_2_O. The qPCR reaction conditions as follows: 95 °C for 30 s, followed by 40 thermal cycles of denaturation at 95 °C for 5 s, annealing at 55–60 °C for 34 s. For each sample, we conducted three biological and three technical replicates. The relative expression level for each gene was calculated as 2^-∆∆CT^ [∆C_T_ = C_T, Target_ - C_T, CYP2_. ∆∆CT = ∆C_T, treatment_ - ∆C_T, CK (0 h)_] [51] compared to that of the untreated control plant which was set as 1 [52]. Statistical analyses were conducted using GraphPad software [53].

## Detailed responses to reviewers

Dear Editor,

Thank you for give me the opportunity to revise my article entitled “ **Comparative genomic analysis of the WRKY III gene family in*populus*, grape,*arabidopsis*and rice**” (MS: **6224766141658131**). We greatly appreciate the concerns and suggestions provided by the editor and two reviewers, and have tried to make our manuscript more clearly by careful correction and already had the language of this paper corrected by a professional scientific editor from ELIXIGEN.We hope that the revised text now is suitable for your journal. The detailed replies to each reviewer will be outlined one by one as follows.

Thank you very much for your consideration. In the case of any questions, please feel free to contact me.

Sincerely yours

Yan Xiang

### Response to reviewer 1

The study is well done, in particular, there is a thorough phylogenetic analysis (nj is a nice standard method, and phylogenetic trees were checked by bootstrapping). Of course the authors could use more demanding methods such as parsimony or maximum likelihood for these trees, but probably the results will not change substantially

-->recommendation: explore this somewhat (alternative calculation by parsimony or ML) and tell the reader the outcome.

***Response:****The reviewer raised a professional and valuable suggestion. Since the NJ tree was extensively used to examine the phylogenetic relationships in the current gene family analysis, and in many reported studies, phylogenetic relationship analyses were initially constructed with neighbor-joining (NJ) method in this study, and the results were completely consistent with previously studies (Jiang et al. 2014). According to the reviewer’s suggestion, and we also constructed a phylogenetic tree from alignments of the full-length sequences of Arabidopsis, rice, grape and Populus WRKY III proteins using maximum parsimony method. The phylogenetic analysis based on Maximum Parsimony (MP) tree was largely consistent with the phylogenetic relationship of the NJ tree.*

Furthermore, conserved motifs (MEME server), gene expression and microsyntheny were examined including reporting of all new experimental data and determination of orthologues and paralogues. WRKY III genes in rice, grape, *Arabidopsis* and *Populus*, were analyzed including their exon-intron structures as well as Ka/Ks analysis and identifying selection pressures on different domains. In addition, the gene expression was compared for different drought stresses (ABA, PEG) and differental expression changes between WRKY III genes determined (eg. *PtrWRKY62, −54, −64, −63, −30* and −*41* were all strongly triggered 9 h after ABA expression).

-->well done and technical sound data provided also in sufficient detail to allow replication of the findings.

-->you should give also a table with functional domain (eg SMART tool) and motif analysis (PROSITE) to get a little bit more insight into the function of the different WRKY III genes (any info on cellular compartment, pathway involvement, differences in molecular function?), the listing of motifs regarding MEME is there not so informative as including specific functional motifs.

***Response:****The reviewer raised a good and professional suggestion. According to the reviewer’s suggestion, we have searched the specific functional motifs and added the function domain to Additional file **1**: Table S1. To the function of the different WRKY III genes, we searched the Gene Ontology (GO) Darabase, which provides a varity of functions annotation for the 57 WRKY III protein sequences. The detailed information is show in Additional file **2**: Table S2, and corresponding contents were added on the Page 6.*

Discussion

Previous work on 10 WRKY III genes in *Populus* trichocarpa is discussed and the new findings regarding SA and in particular ABA and PEG as draught stressor made clear.

-->make sure that you give a little bit more overview on previous work on WRKY III genes (not only on this species) so that the reader better understands which functions are in stock for the WRKY III genes investigated.

***Response:****This is a very good suggestion. Following the reviewer’s suggestion, we have gave more overview on previous work on WRKY III genes, and compared with our work, described on the Page 15–16 of the “****Discussion****”.*

**Quality of written English:** Acceptable

### Response to reviewer 2

A general problem with this work is that many results are reported for which no relevant information is deduced. Often, results are described in detail that we can just see in figures. The manuscript should be trimmed down to describe only results to which the authors can attach biological relevance.

***Response:****This is a very good suggestion. We have trimmed the manuscript down to describe results in the revised manuscript.*

See for example the first section in results in page 4. Unless these results are used to say something, all the values presented are irrelevant and should not be detailed in the text. Please simplify to a small paragraph with main observations. Same thing with the next section in page 4. We see what is said there in the figure. Only salient points of figures should be commented in the text if they are used to support a relevant deduction.

***Response:****According to the reviewer’s suggestion, we have simplify the first section “Chromosomal distribution and physical properties of WRKY III family in four species genomes” and the second section “Phylogenetic analysis of WRKY III genes in rice, grape, Arabidopsis and Populus” to a small paragraph with main observation in results on Page 4–5.*

Similarly, in page 6 the whole paragraph starting with “To explore the evolution of the genetic relationship within each species, we first analyzed the relationship of the WRKY III genes within each intraspecies. The detailed information is listed in Table S3 (a–d).” should be simplified to a couple of sentences. The current text describe things that we can just see and that don’t need explanation.

***Response:****Following the reviewer’s suggestion, we have simplified the whole paragraph to a couple of sentences in the revised manuscript (Page 6–7). Per your valuable suggestions, we have tried every effort to modify the discussion more rigorously by careful correction.*

Many paragraphs in the results section start with sentences like “To gain further insights into the evolution… ”, “To further obtain exon gain/loss information…”, “To better understand the similarity and diversity of motif compositions…”, which are weak motivations, specially when the following text does not bring the insight or understanding promised and only reports data. For example, at the end of one of these “Motif 9 was unique to the proteins in clade 2 and other unique motifs were found in clade 3.” Yes, we can see this, but why is this important? What do we learn from this?

***Response:****Many thanks for this comment. We realized that our description about the results section start with sentences might not be suitable. And we have changed as followed:*

1. *“To gain further insights into the evolution… ” to “It is well known that gene structural diversity drives the evolution of multigene families. To better understand the structural diversity of WRKY III genes, we generated exon/intron organization maps from the coding sequences of each WRKY III gene. The details structural analysis of the exon/intron were presented in Fig. **3**. The 57 WRKY III genes contain different numbers of exons, ranging from 2 to 6.”*
2. *“To further obtain exon gain/loss information…” to “We further analyzed the exon/intron structure of the WRKY III orthologous and paralogous gene pairs that clustered together at the terminal branch of the phylogenetic tree to obtain some traceable information about these genes.”*
3. *“To better understand the similarity and diversity of motif compositions…” to “In addition to the WRKY exon/intron structure, other conserved motifs could be important to the diversified functions of WRKY proteins from rice, grape, Arabidopsis and Populus. Therefore, we used the MEME web server to search the conserved motifs which were shared with the 57 WRKY proteins. A total of 20 distinct conserved motifs were found, and the conserved amino acid sequences and length of each motif are shown in Additional file **1**: Table S1.*”
4. *“Motif 9 was unique to the proteins in clade2 and other unique motifs were found in clade 3.” to “As illustrated in Fig. **4**, most WRKY members within the same clade, particularly the most closely related members, generally shared common motif compositions (e.g. PtrWRKY27 and VvWRKY52), suggesting function similarities among WRKY proteins. Motif 2 is the most common motif, found in all fifty-seven WRKY III genes. Motif 9 was unique to the proteins in clade2 and other unique motifs (e.g. motif 17, motif 18 and motif 19) were found in clade 3; these motifs might be important to the functions of unique WRKY III protein. Motif 7 was mainly present in clade 3 except OsWRKY64 and VvWRKY42, which existed in clade 1 and 2, respectively. To some extent, these specific motifs may contribute to the functional divergence of WRKY genes. The detailed information is shown in Additional file **1**: Table S1.*

Some explanations summarizing results lack content: page 8 “the majority of WRKY III genes are randomly scattered in the genomes” (can the authors support this?); page 10 “As predicted from the basic Ka/Ks analysis, the sliding window analysis clearly showed that numerous sites/regions are under neutral to strong negative or purifying selection.” (what else could be observed?); page 10 “some difference was observed among these genes”; page 11 “most of the genes had a different response to the two treatments (Fig. 11a, b). However, some genes showed similar responses to the two treatments…” (following text to the end of the paragraph non-informative); “Taken together, the similarities in gene structures and motif compositions of most WRKY proteins lend support to the phylogenetic analysis.” (what did the authors expect?); the expression pattern of *PtrWRKYIII* gene was identified to be possibly involved in xylem formation and drought/disease response” (this is not a very concrete conclusion).

***Response:****We sincerely thankful for the careful review from the reviewer. And we made the detailed answer as followed:*

1. page 8 “the majority of WRKY III genes are randomly scattered in the genomes” (can the authors support this?);

*The sentence might be arbitrary and has been removed in the revised manuscript.*

2) “As predicted from the basic Ka/Ks analysis, the sliding window analysis clearly showed that numerous sites/regions are under neutral to strong negative or purifying selection.” (what else could be observed?);

*Following the reviewer’s suggestion, we added more observation on Page 9 (Line 28–30)*.

3) page 10 “some difference was observed among these genes”; page 11 “most of the genes had a different response to the two treatments (Fig. 11a, b). However, some genes showed similar responses to the two treatments…” (following text to the end of the paragraph non-informative);

*page 10: We haved changed the sentence “some difference was observed among these genes” into “The results of SA treatment showed a wide variety of PtrWRKYIII gene expression profiles (Fig. **10b**).*”

*page 11: We haved changed the “most of the genes had a different response to the two treatments (Fig. **11a, b**). However, some genes showed similar responses to the two treatments…” into “Significant expression level changes were observed for 10 PtrWRKYIIIs under the two treatments, of which 8 were up-regulated by PEG treatment, 9 were up-regulated by ABA treatment, however, PtrWRKY90 was down-regulated at different time-points following the two treatments (Fig. **11**). It suggested that more 80 % of the PtrWRKYIIIs analyzed were drought responsive. Examination of the number of PtrWRKYIIIs with significant expression level changed at different time-points of treatment showed that the expression of 6, 1 and 1 PtrWRKYIIIs were changed after PEG treatment for 1, 3 and 24 h, respectively, and the expression of 6 and 3 PtrWRKYIIIs were changed after ABA treatment for 9 and 3 h, respectively (Fig. **11**). It suggested that the majority of PtrWRKYIIIs have altered expression levels at the time-point of 1 h and 9 h under PEG and ABA treatments. Under PEG and ABA trement, only PtrWRKY90 was down-regulated at all time points, which indicating that these genes may play different roles in the response to different drought stresses.”*

4) “Taken together, the similarities in gene structures and motif compositions of most WRKY proteins lend support to the phylogenetic analysis.” (what did the authors expect?);

*The sentence might be confusing and has been changed into “The similarities in gene structure and motif composition of most WRKY proteins consistented with phylogenetic analysis of the WRKY III gene family.”*

5) “the expression pattern of *PtrWRKYIII* gene was identified to be possibly involved in xylem formation and drought/disease response” (this is not a very concrete conclusion).

*Following the reviewer’s suggestion, we have changed this conclusion into “the expression pattern of PtrWRKYIII gene identified that these genes play important roles in the xylem during poplar growth and development, and may play crucial role in defense to drought stress.”*

* Detailed comments.

“WRKY III genes, which are the most advanced and successful in terms of evolution and adaptability”. This is unfunded. Please remove.

***Response:****Many thanks for this comment. The reviewer raised a professional and valuable suggestion. We have removed this sentence in the revised manuscript.*

The second paragraph in the introduction starting with “The WRKY III family has been studied phylogenetically…” repeats the first one. They should be merged.

***Response:****We believe that the reviewer’s suggestions are reasonable, and we have merged it with the first one in the revised manuscript.*

The sentence starting with “In most comparative genomic analyses, three representative lineages of flowering plant species are incorporated…” until the end of the paragraph is disconnected and would fit better in the next paragraph.

***Response:****Many thanks for this comment. We accepted this question sincerely, and we have modified this part and merged it with the next paragraph in the revised manuscript.*

Regarding the motif analysis in page 5, it would be nice to see the correspondence to the zn-finger domains in the figure. If all these proteins belong to the same family, how it is possible that they don’t share a common motif?

***Response:****Following the reviewer’s suggestion, we have added the zn-finger domains in the Fig. **4**. As shown in Fig. **4**, all these proteins share a common motif 2. Other motifs were not shared by all protein, there were two factors contributing to these phenomenons, one reason is these WRKY III gene sequences were obtained from different references, which existed different standard about definition; the another is the WRKY III domain exited variation. Such as, WRKY domain contains the highly conserved amino acid sequence WRKYGQK, but these seven amino acid sequences were not consistent. Some amino acid members (W, Q and K) can mutant and the Q site has high mutation frequence. In some WRKY genes, the WRKY domain can be characterized as WRRK, WSKY, WKRY, WVKY, or WKKY. (Xie Z et al. 2008)*

In page 7: “Subsequently, to gain insight into the microsynteny relationship of WRKY III genes within interspecies, the 57 WRKY III genes were classified into four distinct clades”. This was already used and explained before in page 4. Please, merge there. In any case, the subsequent detailed explanation can be just seen in the figure and is not needed.

***Response:****According to the reviewer’s suggestion, we have merged there with before, and deleted subsequent detailed explanation on the Page 7.*

In my opinion, all conclusions extracted from Fig. 8 are obvious. “(*PtrWRKY62* and −*89*) revealed sites with higher Ka/Ks ratios (Ka/Ks ratios >1) in their domains, indicating positive selection in this region.” What is the conclusion beyond this? Why is this relevant?

***Response:****Many thanks for this comment. We have added the conclusion beyond this and explained why this relevant is on Page 10 in the revised manuscript.*

*One exception (PtrWRKY62 and −89) revealed sites with higher Ka/Ks ratios (Ka/Ks ratios >1) in their domains, indicating positive selection in this region, and implying these two genes experienced somewhat different selective pressure, which reveals the domains showing a higher evolutionary rate that is otherwise hidden in the average value of the Ka/Ks ratio. In addition, positive selection contributes to a higher Ka/Ks ratio, yet it does not guarantee that the gene-average Ka/Ks ratio is over one. Combining Ka/Ks ratios and a sliding-window analysis, we provided evidence suggesting that negative or purifying selection might have played an important role in the evolution of the WRKY III gene family in Populus.*

“the highest expression level of *PtrWRKY41* was observed at 24 h after SA treatment, while that of *PtrWRKY53* was observed at 9 h.” I think a much more relevant difference here is that one gene is 10xfold up-regulated while the other is only 1.2xfold up-regulated.

***Response:****We accepted this question sincerely, and we have re-written it on Page 11 (Line 10–11).*

“suggested that orthologous genes may have originated from a common ancestor.” I don’t see the connection to the previous sentence. And anyway, orthologs by definition originate from a common ancestor. I don’t understand the next sentence “For two of the orthologous genes pairs from poplar and grape, this difference may reflect the fact…” Which difference?

***Response:****Many thanks for this comment. We really say sorry to the reviewer for our careless, and we have deleted the sentence on Page 12 “suggested that orthologous genes may have originated from a common ancestor”. “For two of the orthologous genes pairs from poplar and grape, this difference may reflect the fact…”, this sentence might exist ambiguity, so we have re-written it on Page 12 (Line 26–29).*

“In the four WRKY III clades, genes from poplar, grape, *Arabidopsis* and rice exhibited high levels of microsynteny, which indicated that the WRKY III genes existed before the divergence of the four genomes (poplar, grape, *Arabidopsis* and rice).” High levels respect to what? And, is not the tree already showing that the genes existed before divergence of those species?

***Response:****Many thanks for this comment. We feel so sorry to the reviewer for our lack of clarity. “High levels” respect to “these genes evolved from a duplication event more recently”. The tree already showing that the genes existed before divergence of those species. But a less definite inference between monocots and eudicots using microsynteny was reasonable and possibly due to the far divergence of monocots and eudicots. WRKY III gene family whose evolutionary relationship cannot be inferred based on the traditional phylogenetic tree analysis. The microsynteny can be used to validate or correct the evolutionary relationships in poorly supported nodes in traditional phylogenetic trees.*

In page 14: “a large amount of microsynteny was detected among poplar, grape and Arabidopsis, and little or no microsynteny between rice and poplar, grape and Arabidopsis”. This is repeated. Also a bit later “*Populus* and grape belong to eurosid I, and the number of WRKY III genes is small; *Arabidopsis* is a eurosid II species, being more distantly related to the other two species [27].” As well as the following sentences until line 15.

***Response:****Many thanks for this comment. We believe that the reviewer’s suggestions are reasonable, and we have re-written this part on Page 13–14 in the revised manuscript.*

“In *Populus*, 10 WRKY genes, belonging to group III were induced by varieties of stresses, such as cold, salinity, SA and drought, but no further analysis was performed [6].” How do those results compare to the results presented here?

***Response:****According to the reviewer’s suggestion, we have compared those results to the results presented here on Page 15 (Line 11–16, Line 19) in this revised manuscript.*

“Paralogs originating from duplication within one organism may have more divergent functions.” First of all, paralogs are by definition originating by duplication in one organism. Secondly, more divergent functions than what?

***Response:****We believe that the reviewer’s suggestions are reasonable. We have explained more clearly on Page 16 in this revised manuscript.*

Figures 9, 10 and 11. Since the y-axis scale is different in each graph, it would help to have horizontal discontinuous lines marking the 1.0 value.

***Response:****This is a very good suggestion. We have added horizontal discontinuous lines marking the 1.0 value in the Fig. **10** and **11*. *But Fig. **9** is different from 10 and 11, which reflect that numerous sites/regions are under neutral to strong negative or purifying selection, only two gene pairs were more than the 1.0 value, so we cannot mark the 1.0 value as a standard.*

In Fig. 10 it would be nice to have the same gene order in panels a and b.

***Response:****We agree with the reviewer and have changed the order of these pairs gene in Fig. **10*.

* Minor points

“Each WRKY domain contains a C-terminal located novel zinc finger”. Novel?

***Response:****Many thanks for this comment. In the revised manuscript, we have changed “Each WRKY domain contains a C-terminal located novel zinc finger” into “Each WRKY domain contains a zinc finger motif at the C-terminus”.*

“Temporal expression analysis of group III members in A. thaliana supported the view that these members are part of different plant defense signaling pathway, including compatible, incompatible, and non-host interactions [20]” Ref 20 is about human influenza virus A. Is this really related?

***Response:****Many thanks for this comment. We really say sorry to the reviewer for our careless. We have reword the related citation in revised manuscript. ([20] Kalde M, Barth M, Somssich IE, Lippok B. Members of the Arabidopsis WRKY group III transcription factors are part of different plant defense signaling pathways. Molecular Plant-Microbe Interactions. 2003;16(4):295–305.)*

“a study of the origin and evolution of WRKY III genes in poplar would be useful to reveal the evolution relationship in this gene family.” This is not a very convincing motivation.

***Response:****Many thanks for this comment. The reviewer gave us a valuable suggestions. We have reword it to “Therefore, a study of poplar WRKY III genes would be useful to understanding the important biological functions of these genes”.*

“Microsynteny has been investigated across several plant species using whole-genome sequences to infer the location of homologous genes (orthology or paralogy).” Can the authors support this with one reference?

***Response:****Many thanks for this comment. In the revised manuscript, we have provided the related citation in revised manuscript.*

*[22]. Li Z, Jiang H, Zhou L, Deng L, Lin Y, Peng X et al. Molecular evolution of the HD-ZIP I gene family in legume genomes. Gene. 2014;533(1):218–28. doi:*10.1016/j.gene.2013.09.084*.*

*[23]. Lin Y, Cheng Y, Jin J, Jin X, Jiang H, Yan H et al. Genome Duplication and Gene Loss Affect the Evolution of Heat Shock Transcription Factor Genes in Legumes. PloS one. 2014;9(7):e102825.*

“the anchor point were ligatured with the best non-self match”. I cannot understand this sentence.

***Response:****Many thanks for this comment. Two regions were considered to have originated from a large-scale duplication event when five or more protein-coding gene pairs flanking the anchor point were ligatured with the best non-self match. “the anchor point were ligatured” means that anchor genes (the WRKY III genes of the four species) in two sections were ligatured, “the best non-self match” means that all genes except itself in two sections by pairwise comparisons to attain the best match with E-Value evalution. This sentence quoted from the related citation, for example:*

*[36]. Feng L, Chen Z, Ma H, Chen X, Li Y, Wang Y et al. The IQD Gene Family in Soybean: Structure, Phylogeny, Evolution and Expression. PloS one. 2014;9(10):e110896.*

*[45]. Zhang X, Feng Y, Cheng H, Tian D, Yang S, Chen J-Q. Relative evolutionary rates of NBS-encoding genes revealed by soybean segmental duplication. Molecular Genetics And Genomics. 2011;285(1):79–90. doi:*10.1007/s00438-010-0587-7*.*

“genes flanking n three pairs”. Typo?

***Response:****We corrected this typographical error. The “n” has been deleted in the revised manuscript. In addition, we really say sorry to the reviewer for our carelessness.*

“Ks values >2.0 because of the risk of saturation”. Can the authors explain a bit more? Saturation of what?

***Response:****Many thanks for this comment. Because higher Ks values are associated with a large degree of uncertainty, thus Ks values = 2.0 was suggested as saturation. (Blanc G, Wolfe KH. 2004; Tang H et al. 2008)*

“first-stand cDNA was synthesized”. Typo? “first-strand”

***Response:****We really say sorry to the reviewer for our careless. We corrected this typographical error. The “ first-stand ” has been corrected to “first-strand”.*

Figure 3 caption. Simplify “Intron phases 0, 1, and 2 are indicated by the numbers 0, 1 and 2, respectively” to “Intron phases 0, 1, and 2 are indicated”

***Response:****Many thanks for this comment. We really say sorry to the reviewer for our careless. Accoding to the Fig. **3**, this sentence have been removed in the Fig. **3** caption.*

**Quality of written English:** Needs some language corrections before being published

## Additional files

Additional file 1: Table S1. Detailed information about the 20 motifs in WRKY III proteins of poplar, grape, *Arabidopsis*, rice. (XLS 36 kb) Additional file 2: Table S2. The function prediction of WRKY III genes of *Populus*, Grape, *Arabidopsis*, Rice. (XLS 169 kb) Additional file 3: Table S3. Synteny data in *Populus*, Grape, *Arabidopsis*, Rice, Clade1, Clade2, Clade3, Clade4. (XLS 95 kb) Additional file 4: Table S4. List of primer sequences used for qRT-PCR analysis of the 10 poplar WRKY III genes. (XLS 30 kb)

## Abbreviations

qRT-PCR: Quantitative real-time reverse transcription PCR
TF: Transcription factor
NJ: Neighbor-Joining
MP: Maximum Parsimony
Ks: The rate of synonymous substitutions
Ka: The rate of nonsynonymous substitutions
PEG: Polyethylene glycerol
SA: Salicylic acid
ABA: Abscisic acid
